# Physics-Informed Neural Networks with Unknown Partial Differential Equations: An Application in Multivariate Time Series

**DOI:** 10.3390/e27070682

**Published:** 2025-06-26

**Authors:** Seyedeh Azadeh Fallah Mortezanejad, Ruochen Wang, Ali Mohammad-Djafari

**Affiliations:** 1 School of Automotive and Traffic Engineering, Jiangsu University, Zhenjiang 212013, China; azadeh.fallah@ujs.edu.cn; 2 International Science Consulting and Training (ISCT), 91440 Bures sur Yvette, France; djafari@ieee.org; 3 Shanfeng Company, Shaoxing 312352, China

**Keywords:** physics-informed neural network (PINN), Bayesian computation, partial differential equations (PDEs), multivariate time series (MTS)

## Abstract

A significant advancement in Neural Network (NN) research is the integration of domain-specific knowledge through custom loss functions. This approach addresses a crucial challenge: How can models utilize physics or mathematical principles to enhance predictions when dealing with sparse, noisy, or incomplete data? Physics-Informed Neural Networks (PINNs) put this idea into practice by incorporating a forward model, such as Partial Differential Equations (PDEs), as soft constraints. This guidance helps the networks find solutions that align with established laws. Recently, researchers have expanded this framework to include Bayesian NNs (BNNs) which allow for uncertainty quantification. However, what happens when the governing equations of a system are not completely known? In this work, we introduce methods to automatically select PDEs from historical data in a parametric family. We then integrate these learned equations into three different modeling approaches: PINNs, Bayesian-PINNs (B-PINNs), and Physical-Informed Bayesian Linear Regression (PI-BLR). To assess these frameworks, we evaluate them on a real-world Multivariate Time Series (MTS) dataset related to electrical power energy management. We compare their effectiveness in forecasting future states under different scenarios: with and without PDE constraints and accuracy considerations. This research aims to bridge the gap between data-driven discovery and physics-guided learning, providing valuable insights for practical applications.

## 1. Introduction

NN algorithms are widely used across various fields for a multitude of tasks, often outperforming other methods. Consequently, a diverse range of algorithms has been developed. For instance, there are algorithms designed for spatial relationships in images, sequential data like MTS, and tree-based models demonstrating impressive performance. Most of these algorithms require rich datasets to effectively learn patterns and features for tasks such as regression, classification, or clustering. PINNs, first introduced in [1], improve upon traditional methods by incorporating known physics—specifically PDEs and boundary conditions—into the loss functions as a regularization technique, resulting in more accurate outcomes. One of the key benefits of merging data-driven approaches with fundamental physical principles is their effectiveness in handling small sample sizes and noisy data. Their versatility allows them to be applied across various fields, including fluid dynamics, heat transfer, and structural analysis, where conventional numerical methods often face challenges.

Bararnia and Esmaeilpour [2] explored the application of PINNs to address viscous and thermal boundary layer problems. They analyzed three benchmark scenarios: Blasius–Pohlhausen, Falkner–Skan, and natural convection, specifically investigating the impact of equation nonlinearity and unbounded boundary conditions on the network architecture. Their findings revealed that the Prandtl number plays a crucial role in determining the optimal number of neurons and layers required for accurate predictions using TensorFlow. Additionally, the trained models effectively predicted boundary layer thicknesses for previously unseen data. Hu et al. [3] presented an interpretable surrogate solver for computational solid mechanics based on PINNs. They addressed the challenges of incorporating imperfect and sparse data into current algorithms, as well as the complexities of high-dimensional solid mechanics problems. The paper also explored the capabilities, limitations, and emerging opportunities for PINNs in this domain. Wang et al. [4] explored one application of PINNs in optical fiber communication, proposing five potential solutions for modeling in the time, frequency, and spatial domains. They modeled both forward and backward optical waveform propagation by solving the nonlinear Schrödinger equation. Although conventional numerical methods and PINNs produced similar accuracy, PINNs demonstrated lower computational complexity and reduced time requirements for the same optical fiber problems. Mohammad-Djafari et al. [5] examined the use of PINNs for solving inverse problems, particularly in the identification of dynamic systems. They provided a detailed overview of PINNs and demonstrated their effectiveness through various one-dimensional, two-dimensional, and three-dimensional examples.

B-PINNs are an extension of PINNs that incorporate Bayesian inference to quantify uncertainty in predictions. They leverage the strengths of NNs, physics-based modeling, and Bayesian methods to effectively solve PDEs and other scientific problems. One of the key advantages of B-PINNs is their ability to provide measures of uncertainty, which are crucial for risk assessment and informed decision-making. Liang et al. [6] introduced a higher-order spatiotemporal physics-incorporated graph NN to tackle missing values in MTS. By integrating a dynamic Laplacian matrix and employing an inhomogeneous PDE, their approach effectively captured complex spatiotemporal relationships and enhanced explainability through normalizing flows. Experimental results demonstrated that this method outperformed traditional data-driven models, offering improved dynamic analysis and insights into the impact of missing data.

Since the introduction of PINNs, they have been applied to a wide range of problems across various fields. One of the notable advantages of PINNs is their speed and effectiveness when working with small, noisy datasets. However, a key question arises: how can we apply PINNs when we do not have PDEs readily available for our specific problems? While many physical laws governing phenomena in physics, chemistry, biology, engineering, and environmental science are well established, there are scenarios where no such laws or governing equations (e.g., PDEs) are known. In these cases, applying PINNs becomes challenging because PINNs rely on known physical laws to guide the learning process and achieve precise results with smaller sample sizes, fewer layers, fewer neurons, and fewer training epochs.

For example, consider sociological data, where there are no mathematical equations to describe the percentages of different behaviors or trends in human populations. Similarly, in economics, finance, psychology, or social media analytics, the relationships between variables are often complex and not easily captured by predefined PDEs. In such scenarios, one might aim to use PINNs to inform the network about future predictions in regions where no data are available, enabling the network to make precise forecasts even in data-sparse regions.

However, if no PDEs are known or applicable to the data, using PINNs directly is impossible. This highlights the importance of investigating suitable methods for constructing PDEs from raw data. By identifying governing equations or relationships within the data, we can bridge the gap between data-driven methods and physics-informed approaches, enabling the application of PINNs even in domains where physical laws are not yet defined.

Nayek et al. [7] focused on using spike-and-slab priors for constructing governing PDEs of motion in nonlinear systems. The study used Bayesian Linear Regression (BLR) for variable selection and parameter estimation related to these governing equations. Tang et al. [8] introduced an innovative approach for solving nonlinear robust optimization problems. It combines PINNs and PDE construction within an interval sequential linear programming framework. The method efficiently estimates the sensitivities of uncertain constraints using approximate partial derivatives. These estimates are then used to build deterministic linear models. Tang et al. [9] presented a possibility-based solution framework, known as the equivalent deterministic method, to address interval uncertainty in design optimization. The approach involved constructing PDEs and leveraging PINNs to efficiently model the relations between uncertain variables and the system’s responses. Niven et al. [10] introduced a Bayesian maximum a posteriori framework for identifying dynamical systems from time series data. They provided a comprehensive method for estimating model coefficients, quantifying uncertainties, and selecting models while comparing with joint maximum a posteriori and variational Bayesian approximation against the established Sparse Identification of Nonlinear Dynamics (SINDy) algorithm. The results highlighted the benefits of Bayesian inference in identifying complex dynamical systems and provide a robust metric for model selection.

In this paper, we investigate methods for constructing PDEs from raw datasets. The idea is to use a substantial amount of historical data to construct appropriate PDEs, more precisely to provide a large family of parametric PDE family and use methods of model selection. Once developed, these equations can then analyze new, short-term data to forecast future trends. In summary, we use the historical large data bases to construct a PDE model, which can be used for a much smaller NN with new data using the PINN approach. We explore several techniques, including SINDy, Least Absolute Shrinkage and Selection Operator (LASSO), Bayesian LASSO (B-LASSO), surrogate NNs, and Symbolic Regression (SR). Our focus is on constructing suitable PDEs for an MTS analysis and applying PINNs, B-PINNs, and PI-BLR for a regression task. This approach is particularly applicable when dealing with raw data that lack additional information about their source or underlying dynamics. We then compare the results with and without the constructed PDEs to evaluate any improvements in prediction accuracy for the MTS.

Constructing PDEs is particularly important in the context of MTS analysis. Real-world systems—such as climate patterns, financial markets, and industrial sensors—produce complex, interrelated data that evolve over time. Traditional models often struggle to capture both the hidden physical processes and the intricate relationships among variables. By directly learning PDEs from historical data using methods like SINDy or surrogated NNs, we can uncover the underlying forward model that governs the system. We then integrate these equations with new short-term data using PINNs to enhance our predictions. This approach effectively bridges the gap between the interpretability of physics-based models and the flexibility of Machine Learning (ML), enabling us to forecast multivariate systems even when historical data is noisy or incomplete.

This work introduces a novel methodology for constructing PDEs from raw historical data and integrating them with NNs to enhance MTS forecasting with the new data. The main innovation is the development of a framework that combines data-driven PDE discovery with physics-informed ML, providing more accurate and interpretable models for complex systems. Furtherer, this paper aligns with the aims of the special issue on Bayesian Hierarchical Models with Applications by exploring and enhancing hierarchical Bayesian approaches within the context of physics-informed ML. We focus on developing and interpreting models such as B-LASSO, PI-BLR, and B-PINNs. Our contributions involve integrating domain knowledge through hierarchical priors and providing structured uncertainty quantification, thereby exemplifying the versatility and relevance of Bayesian hierarchical modeling in scientific ML—an overarching theme of this special issue. The main contributions of this paper are outlined as follows: In Section 2, we explain the basic assumptions underlying PDEs. Section 3 presents various methods for constructing PDEs from raw datasets. In Section 4, we provide a brief overview of PINNs through the used PINN algorithm in this paper. Section 5 discusses Bayesian versions of PINNs in conjunction with a PI-BLR model. A real MTS dataset is analyzed in Section 6 using the methods presented in this paper. Finally, insights from the article are discussed in Section 7.

## 2. Differential Equations

Ordinary Differential Equations (ODEs) and PDEs are mathematical models that involve unknown functions and their derivatives, which provide quantitative descriptions of a wide range of phenomena across various fields, including physics, engineering, biology, finance, and social sciences.

An example of a first-order ODE is $\frac{dy}{dt}=ky$, where *y* is a function of the single independent variable *t* and *k* is a constant. This equation describes exponential growth or decay.

A well-known second-order PDE is the heat equation: $\frac{\partial T}{\partial t}=k\frac{\partial^{2}T}{\partial x^{2}}$, where T(x,t) represents the temperature at spatial location *x* and time t, with *x* as the spatial independent variable and t as the time variable [11].

Dynamic systems refer to phenomena that evolve over time according to specific rules or equations, as illustrated by the two examples above. These systems can be linear or nonlinear, deterministic or stochastic, and they are crucial for understanding complex behaviors in various applications, such as climate modeling, population dynamics, and fluid mechanics.

Peitz et al. [12] introduced a convolutional framework that streamlines distributed reinforcement learning control for dynamical systems governed by PDEs. This innovative approach simplifies high-dimensional control problems by transforming them into a multi-agent setup with identical, uncoupled agents, thereby reducing the model’s complexity. They demonstrated the effectiveness of their methods through various PDE examples, successfully achieving stabilization with low-dimensional deep deterministic policy gradient agents while using minimal computing resources.

## 3. Methods of PDE Construction

In this section, we explain several methods that are useful for constructing PDEs from raw datasets. Some of these methods, such as SINDy, LASSO, and SR, are well-established methods that share common principles; however, they are not always successful across all case studies. Alternative surrogate NNs employing a different approach can be used to generate PDEs, but it is crucial that the NNs perform well on the unobserved test set to ensure that its derivatives are reliable for building PDEs.

Regardless of the data type, surrogate NNs are highly generalizable to any MTS dataset. As outlined in Appendix E and Appendix F, the process involves directly constructing equations from the data without imposing additional conditions. The approach begins with training an efficient NN on large volumes of historical data to achieve high predictive accuracy. Derivatives are then computed from the learned network, and equations are formulated between derivatives and variables that exhibit strong correlations. These PDEs are subsequently used for short-term forecasting with PINNs. Because this method does not rely on specific assumptions about the data, it can be readily applied across various domains, including finance, epidemiology, sociology, etc.

In this study, we choose Convolutional Neural Network (CNN) and Temporal Convolutional Network (TCN) architectures for the surrogate NNs due to their accuracy in predictions. The constructed PDEs are local approximations and therefore are not considered true PDEs. A larger dataset can enhance the accuracy of these approximations, extending their validity to a broader range of conditions and potentially revealing finer-scale features.

However, there are no guarantees that the approximation converges to the true underlying PDE, if such a PDE exists. Even with a large training set, the approximation may still overlook important aspects of the system’s behavior or overfit to noise in the data. Thus, while a larger dataset is advantageous, it cannot transform a local approximation into a globally accurate or true PDE; it can only improve the local approximation.

### 3.1. Sparse Identification of Nonlinear Dynamics (SINDy)

SINDy is a powerful framework designed to uncover governing equations from data, especially in the context of dynamical systems. Its primary goal is to identify the underlying equations that dictate a system’s behavior by using of observed data. This method is particularly beneficial when the governing equations are not known beforehand, which makes it a valuable asset in fields like fluid dynamics, biology, and control systems. SINDy, introduced in [13], employs sparse regression alongside a library of candidate functions. This approach allows it to identify the essential terms in complex system dynamics, effectively capturing the key features needed to accurately represent the data. Appendix A provides a detailed overview of the SINDy algorithm used to construct PDEs.

Fasel et al. [14] introduced an ensemble version of the SINDy algorithm, which improves the robustness of model discovery when working with noisy and limited data. They demonstrated its effectiveness in uncovering PDEs from datasets with significant measurement noise, achieving notable improvements in both accuracy and robustness compared to traditional SINDy methods. Schmid et al. [15] utilized the weak form of SINDy as an extension of the original algorithm to discover governing equations from experimental data obtained through laser vibrometry. They applied the SINDy to learn macroscale governing equations for beam-like specimens subjected to shear wave excitation, successfully identifying PDEs that describe the effective dynamics of the tested materials and providing valuable insights into their mechanical properties.

### 3.2. Least Absolute Shrinkage and Selection Operator (LASSO)

LASSO, introduced in [16], is a robust regression technique particularly effective for constructing PDEs from data, especially within the framework of PINNs. This method is valued for its dual capability of variable selection and regularization, which allows for the most relevant feature identification while mitigating the overfitting risk. LASSO achieves this by incorporating a penalty equal to the absolute value of the coefficients into the loss function. This approach promotes sparsity in the model, effectively reducing the number of variables retained in the final equation. Such sparsity is vital when dealing with complex systems governed by PDEs, as it aids in pinpointing the most significant terms from a potentially large pool of candidate variables derived from the data.

Zhan et al. [17] investigated the physics-informed identification of PDEs using LASSO regression, particularly in groundwater-related scenarios. Their work illustrated how LASSO can be effectively combined with dimensional analysis and optimization techniques, leading to improved interpretability and precision in the resulting equations. Additionally, Ma et al. [18] examined a variant version, sequentially thresholded least squares LASSO regression, within PINNs to tackle inverse PDE problems. Their findings highlighted the method’s ability to enhance parameter estimation accuracy in complex systems. Their experiments on standard inverse PDE problems demonstrated that the PINN integrated with LASSO significantly outperformed other approaches, achieving lower error rates even with smaller sample data. The LASSO algorithm is summarized in Appendix B for better understanding of the concept.

### 3.3. Bayesian Least Absolute Shrinkage and Selection Operator (B-LASSO)

The Bayesian framework is particularly beneficial for dealing with limited data by incorporating prior beliefs about the parameters. B-LASSO, explained in [19], is a statistical method that combines the principles of Bayesian inference with the LASSO regression technique, allowing us to leverage the advantages of both approaches. This method provides uncertainty quantification and is robust against overfitting, particularly in high-dimensional settings. It is useful for regression problems where the number of predictors is large relative to the number of observations or when there is multicollinearity among the predictors. In the Bayesian approach, we treat the model parameters, known as coefficients, as random variables and specify prior distributions for them. In addition to estimating the coefficients, B-LASSO also estimates the hyperparameter λ to control the strength of the LASSO penalty, assigning a prior distribution to λ as well.

Many papers use B-LASSO for regression modeling. For example, Chen et al. [20] utilized the B-LASSO for regression models and covariance matrices to introduce a partially confirmatory (theory-driven) factor analysis approach. They tackled the challenges of merging exploratory (data-driven) and confirmatory factor analysis by employing a hierarchical Bayesian framework and Markov Chain Monte Carlo (MCMC) estimation, showcasing the effectiveness of their method through both simulated and real datasets. In Appendix C, we explain the B-LASSO algorithm that is employed in this paper.

### 3.4. Symbolic Regression (SR)

SR is a sophisticated method used in ML and artificial intelligence to identify mathematical expressions that best fit a given dataset. Unlike traditional regression techniques that rely on predefined models, SR seeks to discover both the model structure and parameters directly from the data. It employs techniques such as genetic programming, NNs, and other optimization algorithms to derive these mathematical expressions such as complex and nonlinear relationships. The process involves generating a variety of candidate functions and using optimization methods to evaluate their performance against the data. This approach can reveal underlying physical laws or relationships that standard regression techniques might overlook, providing valuable insights into the system being studied. However, the search space for potential mathematical expressions can be vast, resulting in high computational costs and longer training times, especially with complex datasets. There is also a risk of overfitting, particularly if the search space is not well regularized or if the dataset is small. Achieving optimal results often requires careful tuning of several hyperparameters. The algorithm we used is detailed in Appendix D.

SR has applications across various fields, including data science for uncovering relationships during exploratory data analysis, engineering for deriving mathematical models of complex systems, biology for understanding biological processes or gene interactions, and finance for modeling market behaviors and predicting trends. Changdar et al. [21] introduced a hybrid framework that merged ML with SR to analyze nonlinear wave propagation in arterial blood flow. They utilized a mathematical model along with PINNs to solve a fifth-order nonlinear equation, optimizing the solutions through Bayesian hyperparameter tuning. This approach led to highly accurate predictions, which were further refined using random forest algorithms. Additionally, they applied SR to discover interpretable mathematical expressions from the solutions generated by the PINNs.

### 3.5. Convolutional Neural Network (CNN)

CNNs are well known for their ability to handle spatial relationships in various types of data, including images, videos, and even speech. They are also effective for MTS data. Typically, one-dimensional convolution is used for MTS, but two-dimensional convolution can be applied when the data has two-dimensional features. One of the key advantages of CNNs is their ability to automatically learn patterns and features from raw data, which reduces the need for manual feature engineering. They achieve this by using filters that share parameters across different parts of the input, resulting in lower memory usage and improved performance. CNNs are capable of learning hierarchical representations, where lower layers capture simple features and deeper layers identify more complex patterns and abstractions. However, they usually require a large amount of training data to perform well, which can be a barrier in some applications. Additionally, CNNs are sensitive to small changes in the input data, such as noise, translation, or rotation, which can impact prediction accuracy. This sensitivity can lead to overfitting, especially when working with small datasets. To mitigate this issue, techniques like dropout, data augmentation, and regularization can be beneficial.

Recent research has explored various versions and combinations of CNNs to enhance ML efficiency across different problems. For instance, Liu et al. [22] introduced an innovative approach for airfoil shape optimization that integrates CNNs to construct and compress airfoil features, PINNs for assessing aerodynamic performance, and deep reinforcement learning for identifying optimal solutions. This integrated methodology successfully reduces the design space and improves the lift–drag ratio while tackling the challenges of high-dimensional optimization and performance evaluation.

One important aspect to consider when using surrogate NNs, such as CNNs and TCNs, for constructing PDEs is that the network must perform well on unseen datasets. This means that the surrogate NN should effectively learn the function that closely approximates the true relationship between the inputs and outputs. Consequently, by differentiating the network’s output with respect to its inputs, we can derive the approximate true PDEs. To construct PDEs, it is beneficial to incorporate dynamic variables as inputs to the network. This allows the network to be differentiated with respect to these dynamic inputs after training, facilitating the construction of PDEs. It is important to note that while the final equations may not always be true PDEs—since they are defined based on the dataset’s domain—they serve as local approximations of the true PDEs. If the training dataset is comprehensive, the final equations closely approximate the true PDEs. The convolutional surrogate NN algorithm is clarified in Appendix E, designed to construct PDEs from data.

### 3.6. Temporal Convolutional Network (TCN)

TCNs are a type of NN architecture specifically designed for trajectory data and powerful in handling temporal information. They utilize causal convolutions, meaning that the output at any given time step depends only on current and past input values. This characteristic makes TCNs particularly well suited for tasks such as MTS forecasting, speech synthesis, and other forms of temporal sequence analysis. By employing dilated convolutions, TCNs can effectively expand their receptive fields, allowing them to capture long-range dependencies without relying on recurrent connections. This design also enables TCNs to process data across time steps in parallel, resulting in faster training and more stable gradients compared to recurrent NNs. However, while TCNs provide faster training, using very large kernel sizes or high dilation rates for long sequences can increase computational costs and memory usage due to the larger number of parameters involved. The choice of kernel size, stride, and dilation is crucial, as these factors can significantly impact performance and should be carefully selected based on the characteristics of the input data. Additionally, TCNs can be prone to overfitting, especially when trained on small datasets, which necessitates the use of regularization techniques to improve generalization.

For instance, Perumal et al. [23] explored the use of TCNs for rapidly inferring thermal histories in metal additive manufacturing. They demonstrated that TCNs can effectively capture nonlinear relationships in the data while requiring less training time compared to other deep learning methods. The study highlighted the potential advantages of integrating TCNs with PINNs to enhance modeling efficiency in complex manufacturing contexts. We provide an overview of the algorithm used for the TCN model of this work in Appendix F.

## 4. Physics-Informed Neural Network (PINN)

PINNs, introduced in [1], represent a powerful technique that integrates data-driven ML with fundamental physical principles, keeping it at low computational cost. Unlike traditional NNs, which rely solely on data for training, PINNs incorporate known physical laws expressed as PDEs and boundary conditions into the loss functions. This integration acts as a regularization mechanism, enabling PINNs to achieve higher accuracy, especially in scenarios with limited or noisy data. By leveraging the underlying physics, PINNs can generalize better and make predictions in regions where data is sparse or unavailable.

Since 2019, numerous research papers have explored PINNs with various NN architectures and applications. For instance, Hu et al. [3] discussed how PINNs effectively integrated imperfect and sparse data. They also addressed how PINNs tackled inverse problems and improved model generalizability while maintaining physical acceptability. This was particularly relevant for solving problems related to PDEs that governed the behavior of solid materials and structures. Additionally, their paper covered foundational concepts, applications in constitutive modeling, and the current capabilities and limitations of PINNs. Appendix G includes the structure of the applied PINNs in this article. One can exclude Steps 3 and 5 of the PINN Algorithm to adapt it into a typical NN.

## 5. Bayesian Computations

Bayesian computational models are well known for their ability to measure the uncertainty of variables and perform effectively on noisy data. However, their high computational cost can be a significant drawback, making them less accessible for researchers with limited resources. In this section, we introduce a Bayesian model, called PI-BLR, and a B-PINN that we utilize in our work.

### 5.1. Physics-Informed Bayesian Linear Regression (PI-BLR)

BLR provides a robust framework for statistical modeling by integrating prior beliefs and accounting for uncertainty in predictions. Given our interest in incorporating PDEs as a negative potential component—explained in Step 5 of Algorithm in Appendix H—in the model named PI-BLR, the simplicity of a linear model makes it easy to derive the necessary derivatives. This approach not only improves predictive performance but also supports more informed decision-making across various fields, including finance and healthcare.

Fraza et al. [24] introduced a warped BLR framework, specifically applied to data from the UK Neuroimaging Biobank. The paper emphasized the benefits of this approach, such as enhanced model fitting and predictive performance for various variables, along with the capacity to incorporate non-Normal data through likelihood warping. This method was less computationally intensive than Normal process regression, as it eliminated the need for cross-validation, making it well suited for large and sparse datasets. Gholipourshahraki et al. [25] applied BLR to prioritize biological pathways within the genome-wide association study framework. The advantages of employing BLR included its capacity to reveal shared genetic components across different phenotypes and enhanced the detection of coordinated effects among multiple genes, all while effectively managing diverse genomic features.

In this subsection, we integrate PDEs into the BLR model to introduce physical insights into the framework called PI-BLR. The algorithm is outlined in Appendix H. For a standard BLR model, simply omit Steps 3, 5, and 6.

### 5.2. Bayesian Physics-Informed Neural Network (B-PINN)

As mentioned, Bayesian methods are particularly robust in the presence of noisy or sparse data. By treating NN weights and biases as random variables, these methods estimate their posterior distribution based on the available data and physical constraints, enabling effective uncertainty measurement. Integrating Bayesian inference into PINNs naturally regularizes the model, helping to prevent overfitting and improve generalization. By providing posterior distributions, B-PINNs offer valuable insights into the uncertainty and reliability of predictions. However, it is important to recognize that Bayesian inference techniques, such as MCMC and Metropolis sampling, can be computationally intensive, particularly when addressing high-dimensional problems.

Yang et al. [26] developed a B-PINN to address both forward and inverse nonlinear problems governed by PDEs and noisy data. Utilizing Hamiltonian Monte Carlo and variational inference, the model estimated the posterior distribution, enabling effective uncertainty quantification for predictions. Their approach not only addressed aleatoric uncertainty from noisy data but also achieved greater accuracy than standard PINNs in high-noise scenarios by mitigating overfitting. The posterior distribution was used to estimate the parameters of the surrogate model and the PDE. The PDE was incorporated into the prior to impose physical constraints during the training process.

There are numerous approaches to transform a PINN into a B-PINN. The paper by Mohammad-Djafari [27] introduces a Bayesian physics-informed framework that enhances NN training by integrating domain expertise and uncertainty quantification. Unlike traditional PINNs which impose physical laws as soft constraints, this approach formalizes physical equations and prior knowledge within a probabilistic framework. The method constructs a loss function that balances three key components: fitting observed data, adhering to physical laws, and regularizing predictions based on prior estimates from a Bayesian perspective.

In this paper, we utilize a BNN and incorporate the constructed PDEs into the data loss function to form a kind of B-PINN. In Appendix I, we explain the B-PINN used here. For the corresponding BNN, we exclude Step 5, which involves the incorporation of PDEs. As described in Algorithm in Appendix I, the inputs and outputs of the network are represented as vectors, allowing for an arbitrary number of variables. Increasing the dimensionality, however, raises the computational cost. For real-time multivariate system analysis, the NN—whether Bayesian or non-Bayesian—is initially trained on historical datasets. Once trained, the network can efficiently produce predictions on streaming data, making it suitable for systems with moderate dynamics. When the system exhibits very high dynamism, it is beneficial to fine-tune a few parameters in the last one or two hidden layers to adapt to the system’s evolving behavior.

## 6. A Real-World MTS Dataset

We utilize the household electric power consumption dataset from [28], which comprises 2,075,259 samples recorded at one-minute intervals over a period of nearly four years. The data span from 16 December 2006, at 17:24 to 26 November 2010, at 21:02. Each sample includes seven measured variables alongside the corresponding date and time. Although the dataset does not specify the exact location where the measurements were taken, it provides detailed insights into the energy consumption patterns of a single household.

We add a dynamic variable to the dataset by calculating the elapsed time in hours for each sample, starting from zero for the first sample using the information in the date and time columns. To gain a better understanding of the data, we calculate the correlations between each pair of variables. We find that two variables have a correlation of exactly one, indicating that they are linear functions of each other. In other words, knowing the value of one variable allows us to determine the exact value of the other using a simple regression model to derive the linear relationship.

The dataset captures the following key electrical quantities, which we named for easier handling. The input variables are designated by *X* indices, while the outputs are represented by *Y* indices. Since this is an MTS, some of the inputs and outputs can be identical. The outputs are generated by incorporating an appropriate shift (lag), such as 30 time units. The variables in this dataset are explained below.

1. **Elapsed Time** $X_{1}$: The time that has passed since the beginning of the data collection period, measured in hours. This variable enables us to analyze trends and patterns in energy consumption over time, facilitating time-based analyses and comparisons.
2. **Global Active Power** $X_{2}$ and $Y_{1}$: The total active power consumed by the household, averaged over each minute (in kilowatts).
3. **Global Reactive Power** $X_{3}$ and $Y_{2}$: The total reactive power consumed by the household, averaged over each minute (in kilowatts).
4. **Voltage** $X_{4}$ and $Y_{3}$: The minute-averaged voltage (in volts).
5. **Global Intensity**: The minute-averaged current intensity (in amperes). This variable is a linear function of Global Active Power, so we omit it from the dataset.
6. _**Sub-metering**1_ $X_{5}$ and $Y_{4}$: Energy consumption (in watt-hours) corresponding to the kitchen, primarily attributed to appliances such as a dishwasher, oven, and microwave.
7. _**Sub-metering**2_ $X_{6}$ and $Y_{5}$: Energy consumption (in watt-hours) corresponding to the laundry room, including a washing machine, tumble-drier, refrigerator, and lighting.
8. _**Sub-metering**3_ $X_{7}$ and $Y_{6}$: Energy consumption (in watt-hours) corresponding to an electric water heater and air conditioner.

Notably, the dataset contains approximately 1.25% missing values, represented by the absence of measurements between consecutive timestamps. We apply first-order spline interpolation to fill in the missing parts. Additionally, ref. [28] explained that the active energy consumed by appliances not covered by the previously mentioned sub-metering systems can be calculated using the below formula based on the available measured variables:

$$\mathrm{Active}\;\mathrm{Energy}\;\left(\mathrm{Wh}\right)=X_{2}\times \frac{1000}{60}-X_{5}-X_{6}-X_{7}.$$

To enhance the effectiveness of ML methods, normalization is recommended to improve the learning process. In this example, we first normalized the dataset and then applied the described method for further analysis.

In the PDE construction phase, the most effective method is the surrogate NN utilizing a TCN architecture. We employ two models and explain the differences between them. Model 1 is a standard TCN with three convolutional blocks, utilizing filters of sizes 64 and 128 and a kernel size of 3. It features residual connections and dropout layers to reduce overfitting, and it is trained using the Adam optimizer. Model 2 enhances this foundation by incorporating dilated convolutions, which allow it to capture long-range dependencies more effectively. With dilation rates of [1,2,4], it can analyze broader contexts without a significant increase in computational cost. Retaining the residual connections and dropout for stability, Model 2 is specifically trained to focus on temporal dependencies, making it more adept at recognizing patterns over extended periods. The CNN model employs a one-dimensional CNN that consists of three convolutional blocks. Each block includes ReLU activation, max pooling, and dropout layers to effectively construct temporal patterns while minimizing the risk of overfitting. The training process utilizes the Adam optimizer along with Mean Squared Error (MSE) as the loss function. All networks are trained for 30 epochs.

The method used in the surrogate NNs involves training the network on a large historical dataset and then utilizing the trained model to perform differentiation. The key aspect is that the networks must make accurate predictions. If they do, we can use the trained network to calculate derivatives. This implies that when the network accurately predicts unseen data, it identifies the unknown function of the input to generate the outputs. Consequently, we can differentiate this network, which represents a mathematical function of the inputs.

**Figure 1:**
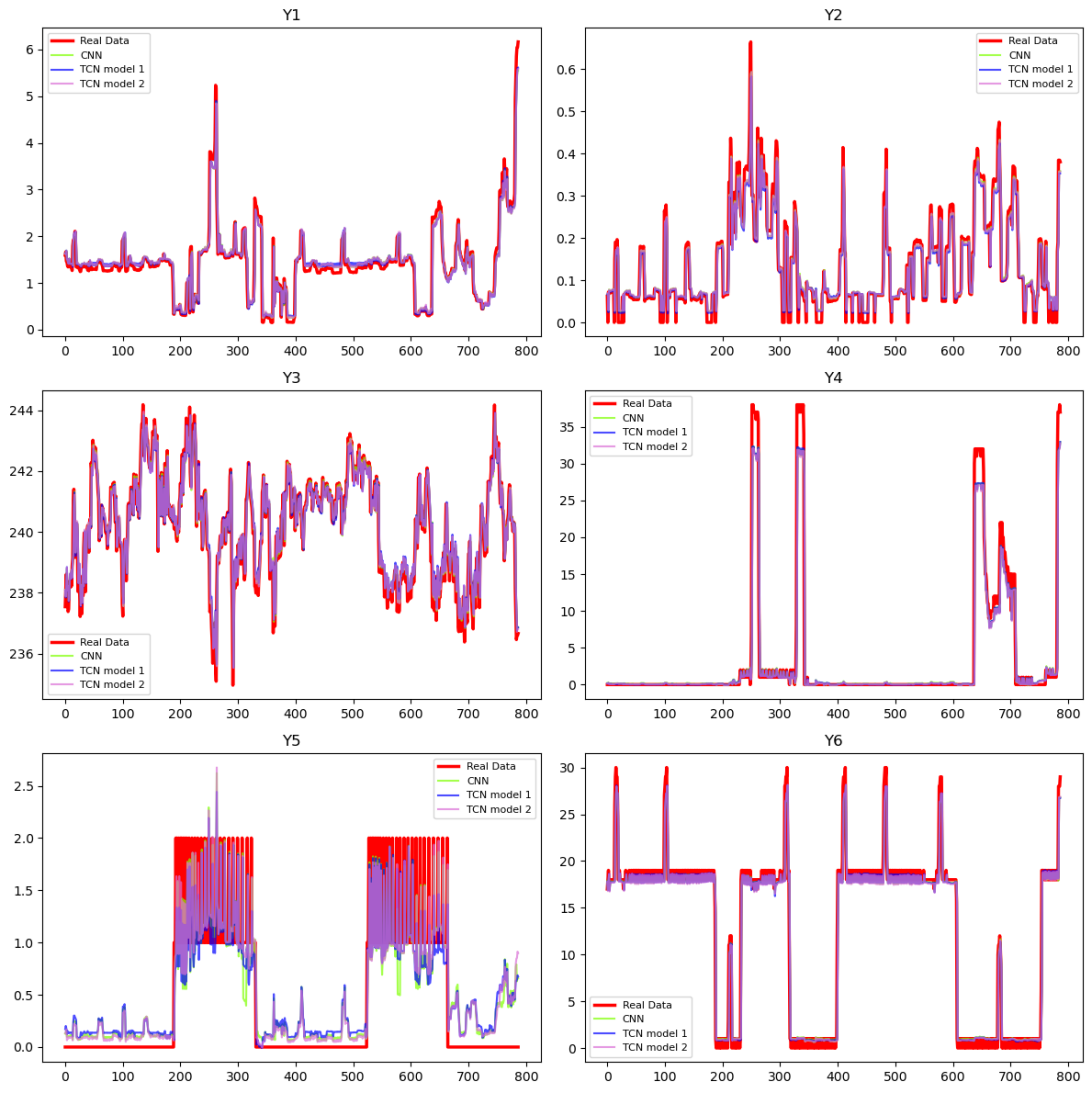
Predictions of the state variables in PDE construction phase via surrogated NNs.

Table 1 displays the metrics of the trained networks, including MSE, Mean Absolute Error (MAE), and the coefficient of determination $R^{2}$, which are used to evaluate predictive performances. Figure 1 displays the corresponding predictions of the surrogate NNs. Based on the results, all three networks make good predictions of the variables, except for $Y_{5}$. As explained earlier, the $Y_{5}$ data relate to the use of certain electrical appliances that are operated intermittently, resulting in many zero values. To evaluate the model’s performance under challenging conditions, we keep the NN simple—comprising three hidden layers and trained for 30 epochs—and observe that, while it performs well for most variables, accurately predicting $Y_{5}$ requires additional computational effort. Our proceeding results demonstrate that the PINN method outperforms traditional NNs, and increasing the network’s complexity has the potential to further enhance this advantage. This setup is employed to assess model performance in more difficult scenarios. The success of these methods heavily depends on the chosen dictionary and various hyperparameters. Based on the evaluation criteria, traditional methods like SINDy, LASSO, B-LASSO, and SR struggle to capture the complex relationships between variables, resulting in PDEs that are less suitable for use in PINNs. In contrast, the PDEs generated from surrogate networks are better suited for integration into the PINN training process.

**Figure 2:**
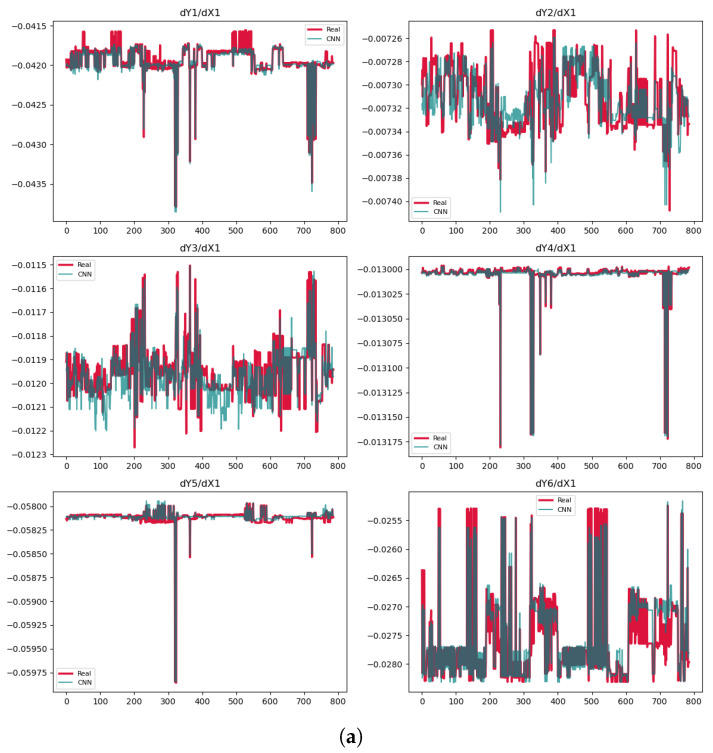

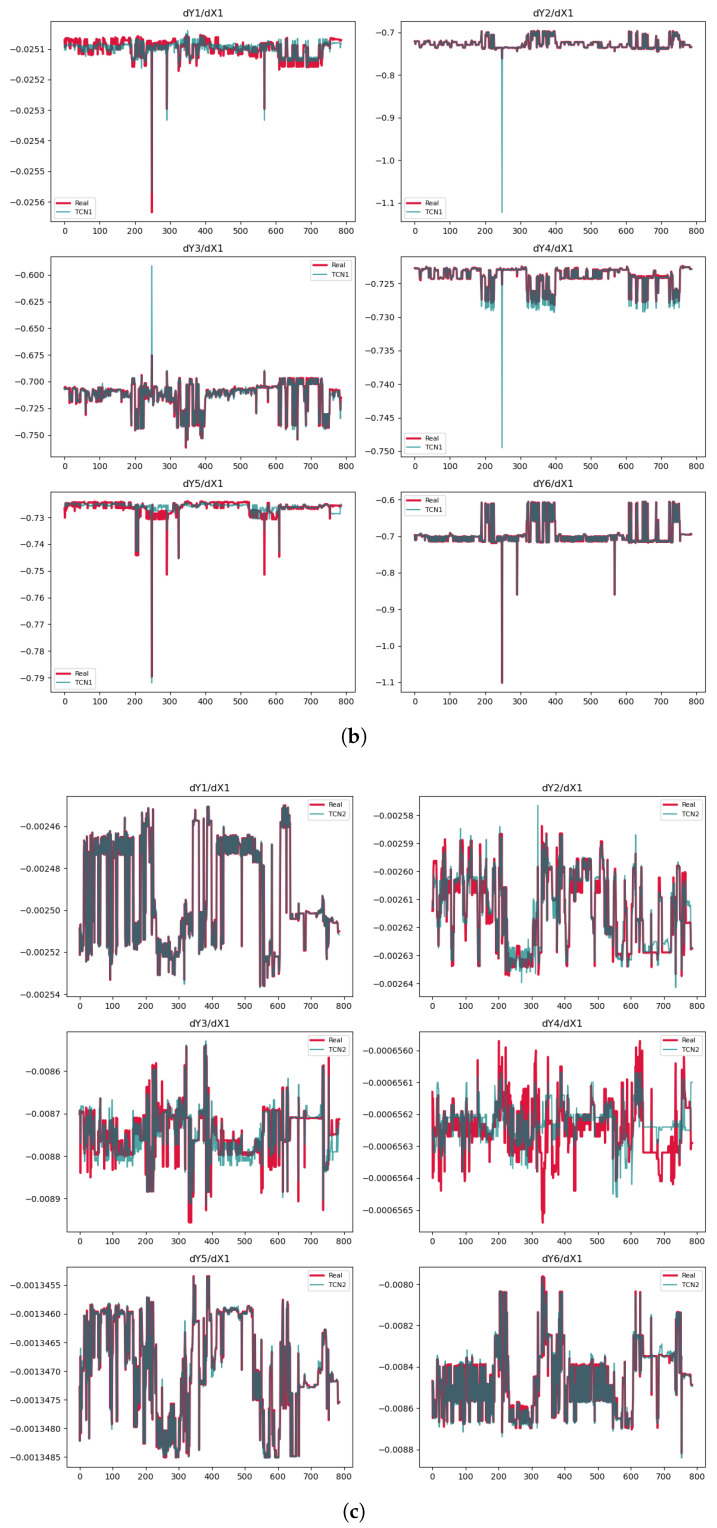
Predictions of the derivatives. (**a**) CNN model. (**b**) TCN Model 1. (**c**) TCN Model 2.

In the next step, we calculate the derivatives of the outputs with respect to all input variables in the train set. Then, we formulate PDEs with respect to the dynamic variable $X_{1}$ using other derivatives based on polynomials of Degree 3. The results of the surrogate NNs, along with those from other methods, are presented in Table 2. Given the highly complex relationships among variables in this example, traditional methods are unable to accurately discover the underlying relations or derive meaningful mathematical equations. Table 2 presents the metrics for all explained PDE construction methods. As indicated by these metrics, traditional approaches—such as SINDy, LASSO, B-LASSO, and SR—produce equations with high error rates, rendering them unsuitable for training NNs. Conversely, the results from surrogate models suggest that these models effectively predict most variations between derivatives and variables. Consequently, we select the most accurate PDEs to train PINN, B-PINN, and PI-BLR, and compare their results with those obtained through learning without explicit PDEs. While Bayesian networks excel in uncertainty analysis, they are computationally intensive; therefore, for quicker computations, PINNs serve as a practical alternative, albeit without uncertainty quantification. Since the metrics indicate significantly large errors for other methods in Table 2, we plot the PDEs only for the surrogate NNs in Figure 2, separately for each network. This is because each NN predicts the outputs using different mathematical functions, resulting in distinct derivatives for each network. We select and prioritize PDEs by first identifying those with the lowest errors and highest $R^{2}$ values, as shown in Table 1 and Table 2. Then, based on visualization of the results in Figure 2, we further shortlist the PDEs that demonstrate better performance.

The $R^{2}$ values are negative or near zero for some cases, indicating poor model performance in Table 2. This occurs because $R^{2}=1-SSR/SST$ becomes negative when the model’s prediction errors (SSR) exceed the natural variability in the data (SST). Such results suggest the model fails to capture meaningful patterns. We only use the PDEs from the TCN models, either Model 1 or Model 2, despite the CNN showing some high values of $R^{2}$. The reason is that the prediction plots in Figure 2a display significant variability, making the derivatives unreliable. In contrast, the predictions for $dY_{2}/dX_{1}$, $dY_{3}/dX_{1}$, and $dY_{6}/dX_{1}$ in Model 1 as well as for $dY_{1}/dX_{1}$ in Model 2 are very accurate. In Model 1, the $R^{2}$ for $dY_{2}/dX_{1}$ is −0.4289, largely due to one part of data that is significantly distant from the exact values.

Since TCNs have a strong architecture for recognizing temporal relationships, we also apply a TCN in the prediction phase. The TCN layer extracts temporal features through dilated convolutions with dilation rates of [1,2,4,8], utilizing 64 filters and a kernel size of 2. The output layer then maps these features to 6 output variables using a dense layer.

We use the same architecture for both PINNs and the standard NNs. The key differences in the PINNs arise from the selection of various PDEs. While the training for PINNs focuses on minimizing (A1), the NNs primarily aim to reduce data loss. For TCN model 1, we develop two distinct PINNs. One of them incorporates the PDE related to $dY_{2}/dX_{1}$, while the other includes PDEs of $dY_{2}/dX_{1}$, $dY_{3}/dX_{1}$ and $dY_{6}/dX_{1}$. We train the non-Bayesian networks for 50 epochs, and to facilitate a better comparison, we include results for early stopping with a maximum of 200 epochs; however, all training terminates in fewer than 50 epochs.

To facilitate comparison across sample sizes in the prediction phase, we select three different sample sizes for the training, validation, and testing procedures. The starting point for the training set is the next data point following the last sample of the PDE construction phase, resulting in distinct sample values. We define small (2000 training, 500 validation, and 500 testing), medium (26,000 training, 4000 validation, and 500 testing), and large (91,656 training, 4324 validation, and 500 testing) sample sizes. The large samples consist of the remaining data set after the PDE construction phase.

**Figure 3:**
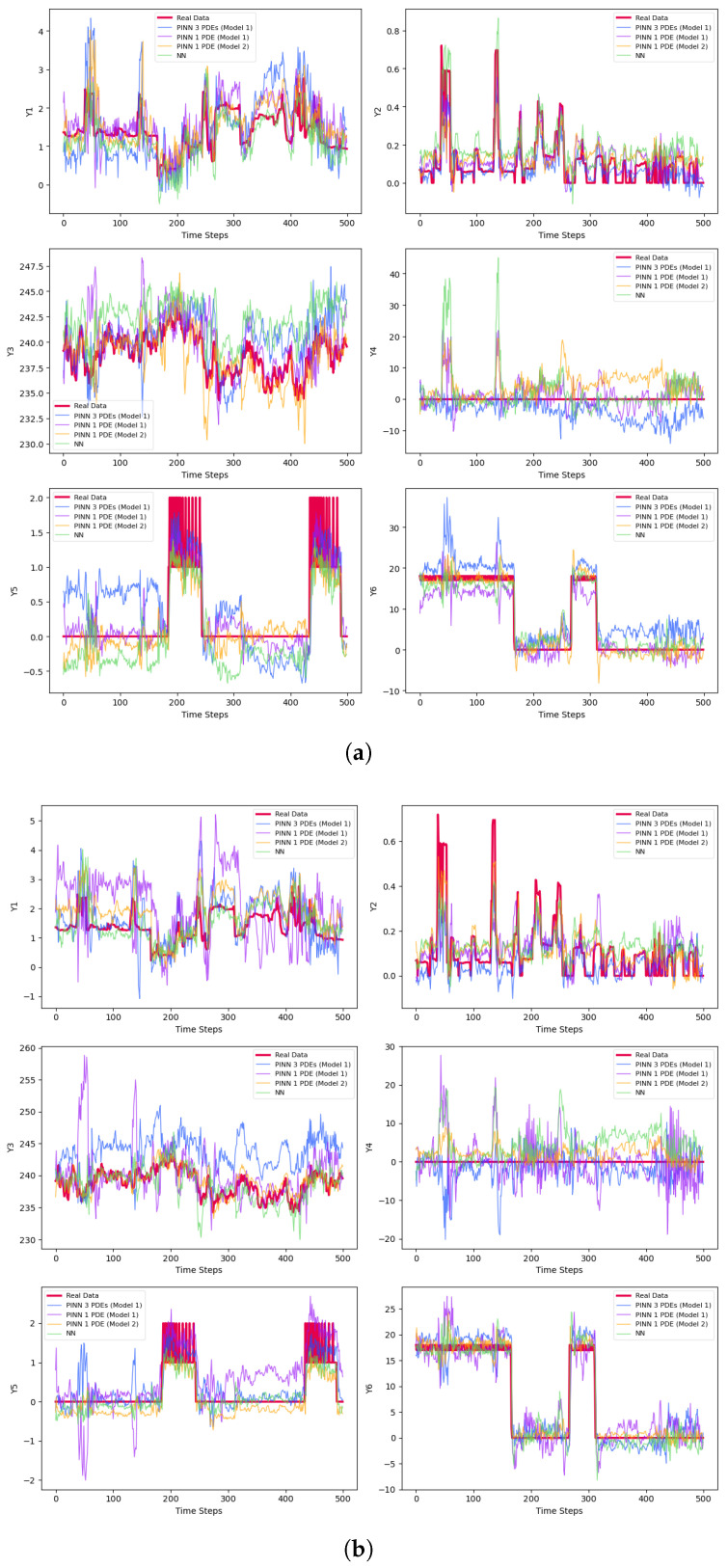

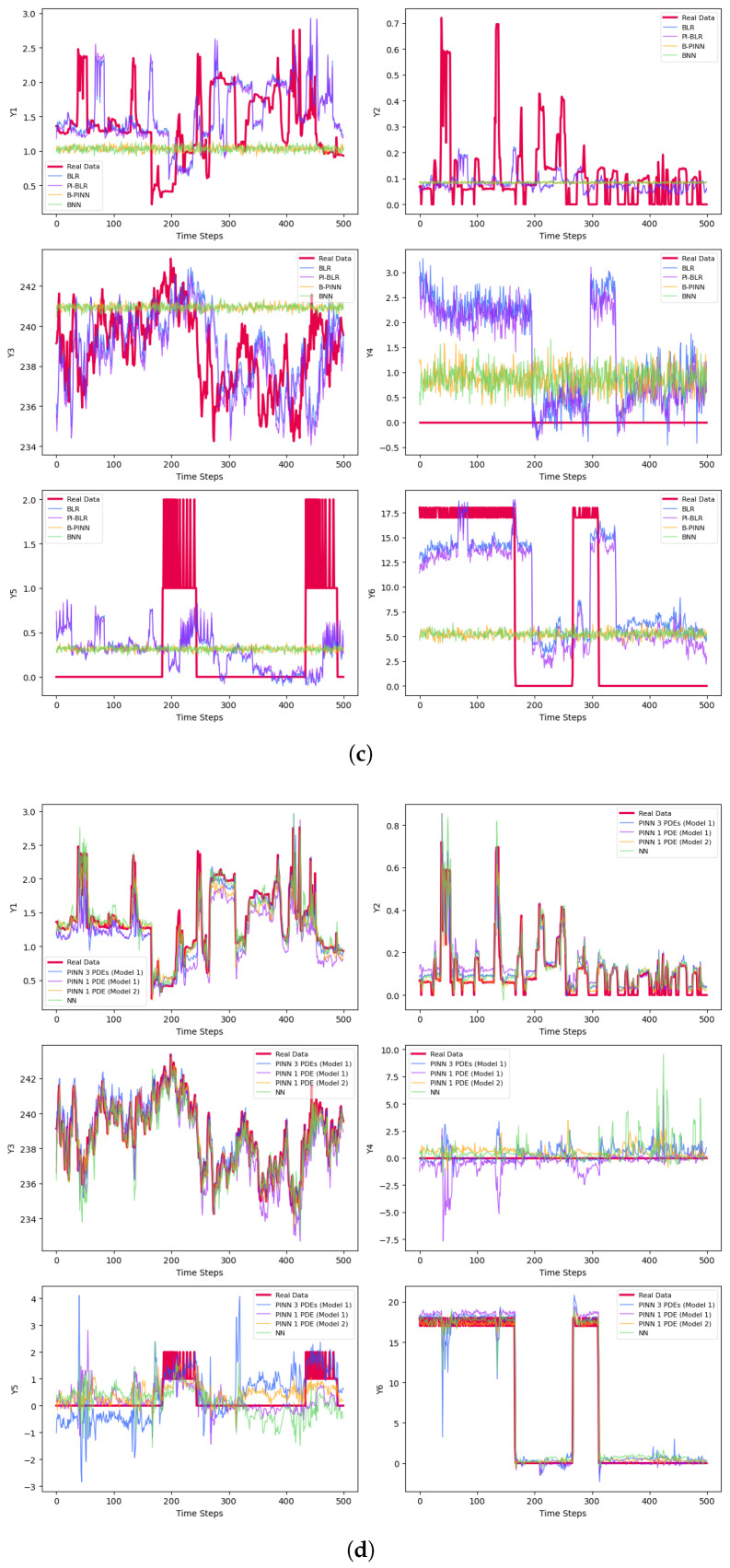

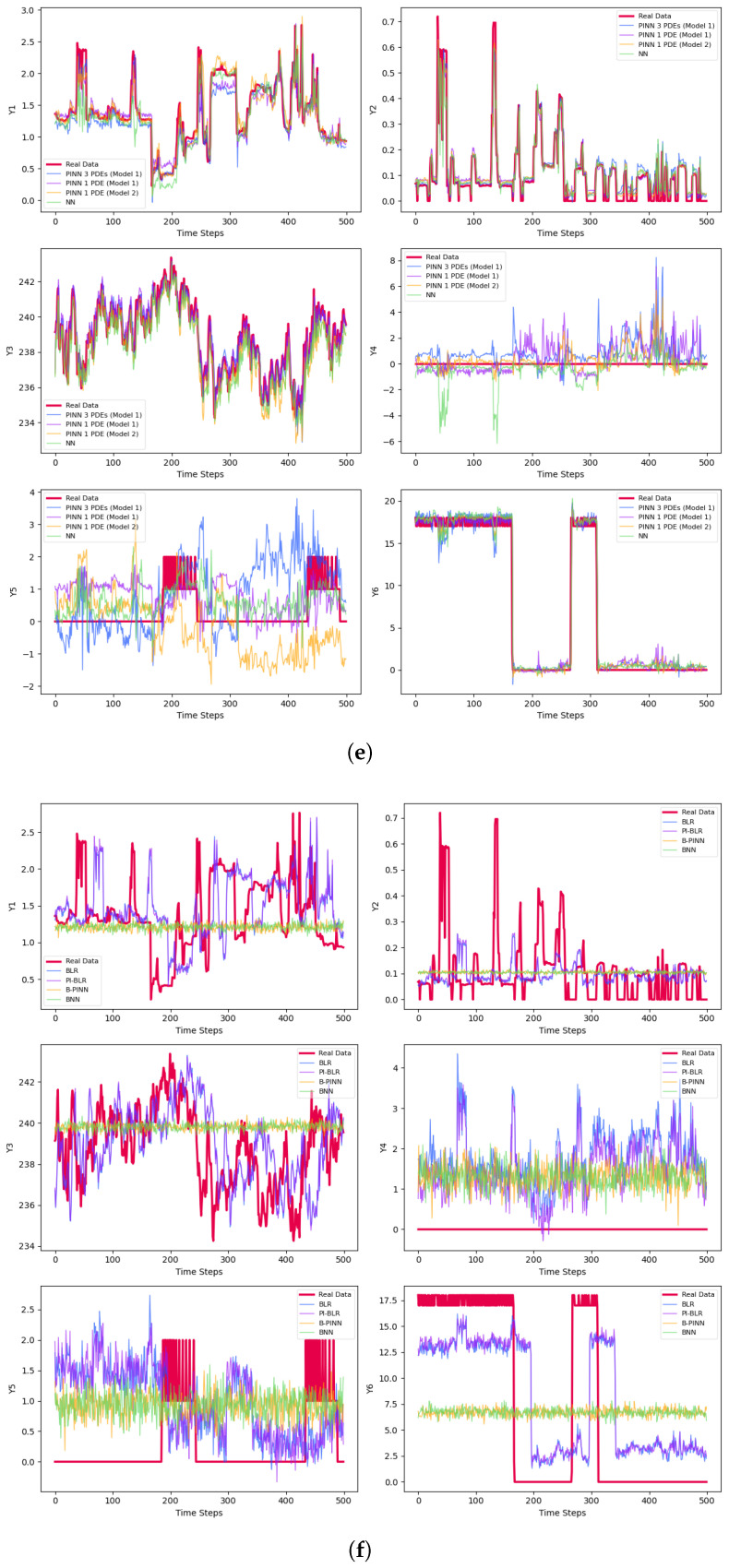

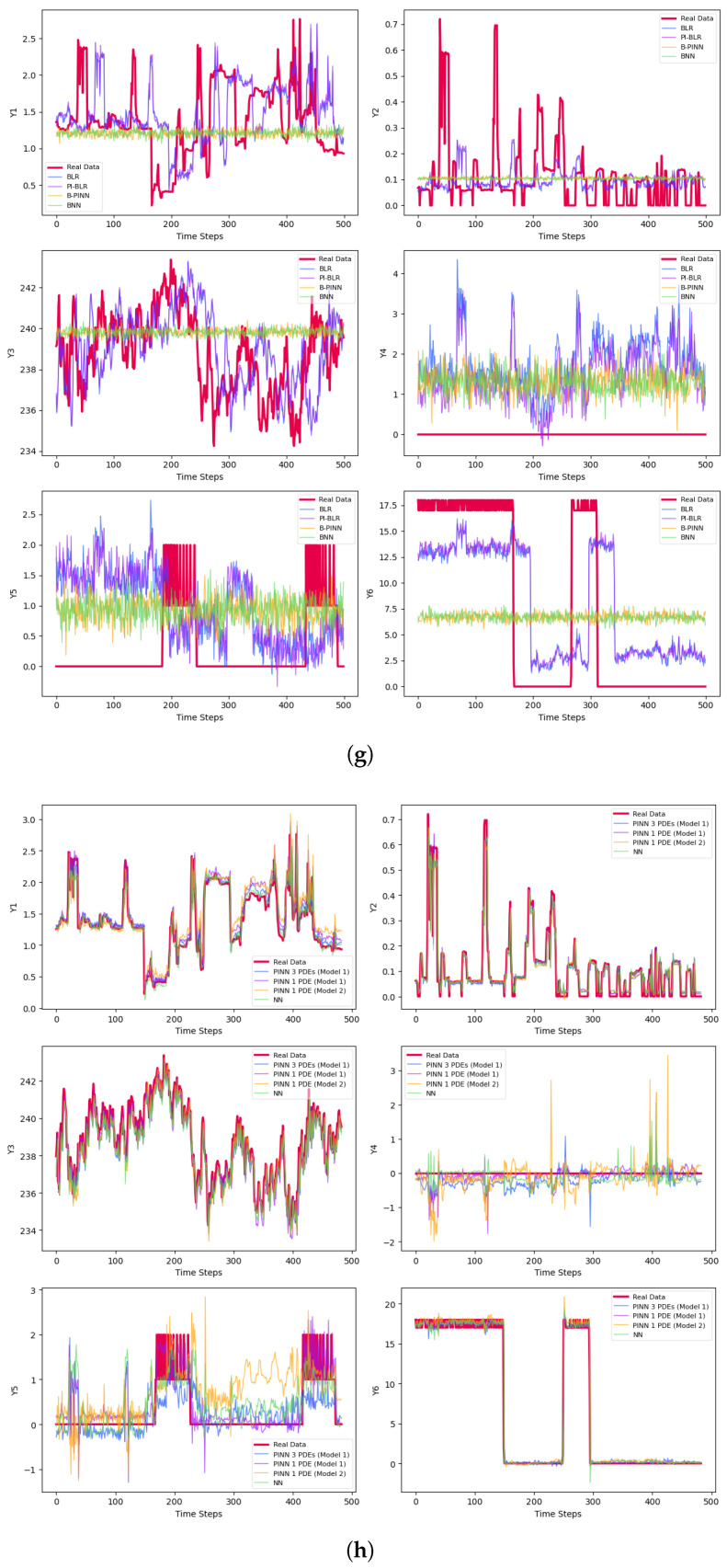

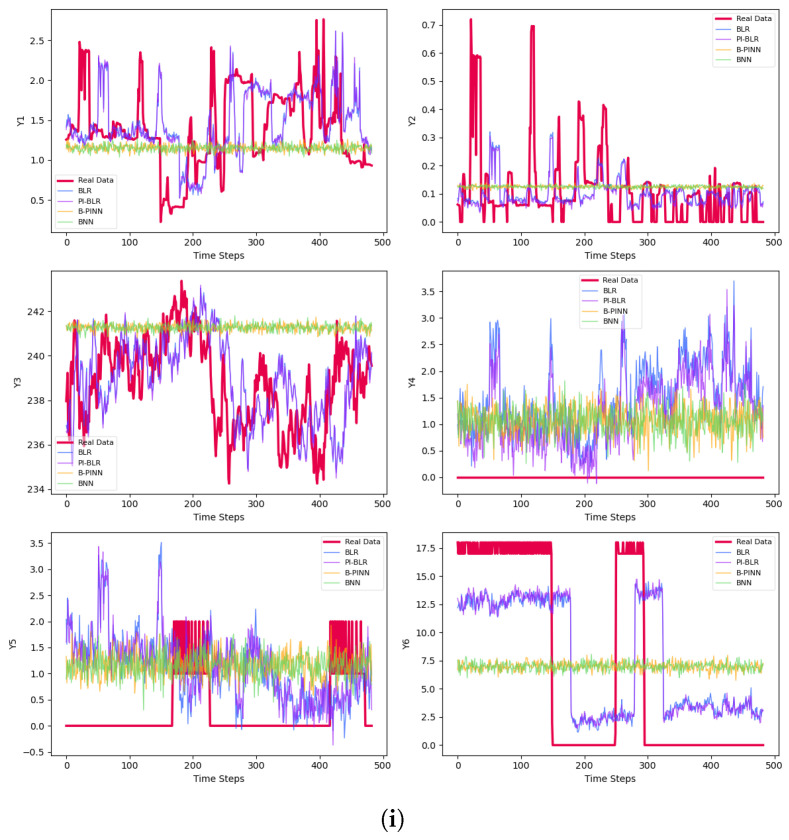
Predictions of the derivatives. (**a**) Predictions of the small samples with 50 epochs. (**b**) Predictions of the small samples with early stopping. (**c**) Bayesian predictions of the small samples. (**d**) Predictions of the medium-size samples with 50 epochs. (**e**) Predictions of the medium-size samples with early stopping. (**f**) Bayesian predictions of the medium-size samples. (**g**) Predictions of the large samples with 50 epochs. (**h**) Predictions of the large samples with early stopping. (**i**) Bayesian predictions of the large samples.

The final prediction results are presented in Table 3 and Figure 3. Figure 3a–c represent results for small batches, while Figure 3d–f correspond to medium-sized samples. The large batch results are shown in Figure 3g–i. For each set, Figure 3a,d,g display the predictions for the test sets after 50 epochs of training. Figure 3b,e,h illustrate the results using early stopping criteria, and Figure 3c,f,i show the prediction curves obtained through Bayesian methods.

Samples with varying sizes are constructed to compare results. Naturally, increasing the sample size improves prediction accuracy on test samples, as more information is fed into the model. However, this is often impractical due to higher costs and longer computation times. A key focus is whether there is a significant performance difference between traditional NNs and the new approach that incorporates physical information—such as PDEs—into the network. While larger sample sizes tend to enhance accuracy, our main comparison is between classic NNs and PINNs. In PINNs, adding physical information increases training time—meaning they take longer to train for the same number of epochs. Interestingly, when PDEs are estimated from data, PINNs can learn effectively with fewer epochs than simple NNs. As the number of training cycles increases, the training time for PINNs also grows, but they can often reach higher accuracy with fewer epochs depending on the application. In this study, we assume PDEs are unknown and derived from historical data, which preserve the advantage of PINNs over traditional NNs that do not leverage PDEs. These equations are particularly useful when relationships between variables are too complex to be captured by simple mathematical models. Estimating PDEs from data generally yields better results than ignoring this information altogether. The following summarized steps outline our comprehensive approach to data preparation and PDE construction for physical information-based networks.

**Data Analysis Algorithm:**

1. Check the correlations between variables and remove those with linear relationships.
2. Use interpolation methods like first-order spline to fill in missing data gaps.
3. Normalize the data to facilitate capturing complex relationships between variables.
4. Divide the data into two portions: approximately 95.35% for the PDE construction phase and 4.65% for the prediction phase using data-driven PDEs. Additionally, split each segment into training, validation, and testing sets.
5. Apply the PDE construction methods outlined in Section 3:
  - The surrogate networks are selected based on their ability to accurately predict on the test samples. Evaluation criteria for surrogate network prediction accuracy are detailed in Table 1 and illustrated in Figure 1.
  - The performance of the PDEs constructed by all methods is evaluated using criteria listed in Table 2. The PDEs obtained from surrogate NNs are shown in Figure 2.
6. Select the most accurate PDEs for training the PINN, B-PINN, and PI-BLR networks, as well as models without physical information. The results are presented in Table 3 and Figure 3.
7. Train the physics-informed network using 4.65% of the remaining samples, along with the data-driven PDEs.
8. Use the previous *T* samples to predict a point in the future. Then, iteratively apply the same process, using the latest predicted values as inputs to generate subsequent future points. Continue this hierarchical prediction until you reach the desired number of future predictions.
9. Evaluate the predictions using metrics such as MSE.

Generally, the non-Bayesian methods perform better and are approximately faster by over 98%. One reason for this is that Bayesian models are more complex and require significantly more samples and chains to train their networks accurately. The limited training time and high computational cost likely contribute to the poor results, which is why we decide to omit the training process for the Bayesian networks when using one PDE from Models 1 and 2. Among the Bayesian methods, the highest $R^{2}$ is achieved by the B-PINN, which utilizes large sample sizes, while the best plot is produced by the BLR and PI-BLR, as shown in Figure 3i.

There are six variables to predict, $Y_{1},\ldots ,Y_{6}$, and three different sample sizes—small, medium, and large—resulting in a total of 18 prediction cases. All networks struggle with predicting $Y_{4}$, consistently yielding an $R^{2}$ of zero across different training sample sizes. For all other variables, except $Y_{1}$, the PINNs produce the best predictions, with the highest $R^{2}$ values highlighted in bold in Table 3. The only exception is $Y_{1}$, which is best predicted by the simple NN. Among the remaining twelve cases, the highest $R^{2}$ values are achieved with TCN Model 2 in nine instances, as shown in Table 3. This superiority mainly stems from Model 2’s use of dilated convolutions, which enhance the network’s ability to capture long-term dependencies and learn more complex data patterns. In TCN Model 2, only one PDE is used. According to Table 2 and Figure 2c, this PDE exhibits high accuracy. For the remaining three cases—namely predicting $Y_{5}$ with small samples, $Y_{2}$ with large samples, and $Y_{5}$ with large samples—TCN Model 1 performs best, utilizing only one PDE. It is worth noting that TCN Model 1 operates in two scenarios: using a single PDE and using three PDEs. The metrics are presented in Table 2 and the prediction plots in Figure 2b. In this example, TCN Model 1, which employs a single PDE, outperforms the same model with three PDEs. Overall, PINNs demonstrate higher prediction accuracy compared to non-physics-based networks. Among the PINN models, TCN Model 2 performs the best, showing advantages in nine prediction cases, followed by TCN Model 1, which uses a single PDE and delivers strong results.

PINNs are well known for providing additional information to the training process, enhancing efficiency. In this research, we do not have predefined PDEs; instead, we construct them from historical data. By incorporating these PDEs, the efficiency of the network improves. In other words, the external computations during the PDE construction phase add valuable information to the network, thereby enhancing its efficiency, even though this information is derived from the available data.

## 7. Conclusions

This work demonstrates that physics-informed learning frameworks can be effectively adapted to systems with unknown governing equations by integrating data-derived PDEs. We show that surrogate TCNs with dilated convolutions can automatically construct meaningful PDEs from raw MTS data. This capability enables the application of PINNs, even in domains where predefined physical laws are absent. While PINNs achieved significant improvements in predictive accuracy for most variables, Bayesian methods revealed trade-offs between computational efficiency and uncertainty quantification. However, Bayesian methods require rich datasets, including sufficient sampling sizes and chains. Interestingly, the constructed PDEs help improve the model’s efficiency by guiding predictions toward physically plausible solutions, even when they are derived only from historical data. These findings highlight the potential of hybrid data-physics approaches in fields such as economics and social sciences, where explicit governing equations are often difficult to identify.

## Figures and Tables

**Table 1:** Performance metrics in PDE construction phase for the state variable predictions.

Phase	Methods	Variable	MSE	MAE	$R^{2}$
PDE constructions 1,958,991 training 19,000 validation 788 testing	CNN	$Y_{1}$	0.0706	0.1383	0.8853
		$Y_{2}$	0.0021	0.0262	0.8483
		$Y_{3}$	0.4367	0.4823	0.8502
		$Y_{4}$	8.6429	0.8770	0.8915
		$Y_{5}$	0.1597	0.2690	0.5696
		$Y_{6}$	3.7938	0.9266	0.9523
	TCN Model 1	$Y_{1}$	0.0711	0.1376	0.8845
		$Y_{2}$	0.0021	0.0269	0.8440
		$Y_{3}$	0.4459	0.4904	0.8471
		$Y_{4}$	8.6066	0.8330	0.8919
		$Y_{5}$	0.1584	0.2762	0.5730
		$Y_{6}$	3.7958	0.9728	0.9523
	TCN Model 2	$Y_{1}$	0.0711	0.1414	0.8844
		$Y_{2}$	0.0021	0.0262	0.8478
		$Y_{3}$	0.4334	0.4813	0.8514
		$Y_{4}$	8.7877	0.8595	0.8896
		$Y_{5}$	0.1516	0.2525	0.5914
		$Y_{6}$	3.8516	0.9888	0.9516

**Table 2:** Performance metrics for PDE construction phase for derivatives. Values in bold indicate the corresponding PDEs that are utilized.

Phase	Methods	Variable	MSE	MAE	$R^{2}$
PDE constructions 1,958,991 training 19,000 validation 788 testing	SINDy	$dY_{1}/dX_{1}$	$5.8788\times 10^{18}$	$2.2649\times 10^{9}$	$-2.7194\times 10^{16}$
		$dY_{2}/dX_{1}$	$7.09188\times 10^{17}$	$7.8661\times 10^{8}$	$-9.41825\times 10^{16}$
		$dY_{3}/dX_{1}$	$5.30668\times 10^{19}$	$6.8055\times 10^{9}$	$-3.32554\times 10^{16}$
		$dY_{4}/dX_{1}$	$2.71052\times 10^{19}$	$4.8655\times 10^{9}$	$-1.14348\times 10^{15}$
		$dY_{5}/dX_{1}$	$8.28482\times 10^{19}$	$8.5026\times 10^{9}$	$-1.61717\times 10^{17}$
		$dY_{6}/dX_{1}$	$8.98478\times 10^{18}$	$2.8000\times 10^{9}$	$-6.38548\times 10^{14}$
	LASSO	$dY_{1}/dX_{1}$	$1.6382\times 10^{3}$	$1.6576\times 10^{1}$	$4.5876\times 10^{-1}$
		$dY_{2}/dX_{1}$	$6.8290\times 10^{3}$	$3.1475\times 10^{1}$	$-2.4088\times 10^{-1}$
		$dY_{3}/dX_{1}$	$2.1774\times 10^{3}$	$2.2091\times 10^{1}$	$1.6338\times 10^{-1}$
		$dY_{4}/dX_{1}$	$6.3249\times 10^{3}$	7.8828	$3.4856\times 10^{-2}$
		$dY_{5}/dX_{1}$	$1.5603\times 10^{2}$	5.1895	$-4.4799\times 10^{-1}$
		$dY_{6}/dX_{1}$	$1.9095\times 10^{4}$	$5.0810\times 10^{1}$	$1.2204\times 10^{-1}$
	B-LASSO	$dY_{1}/dX_{1}$	$1.6237\times 10^{7}$	$2.1261\times 10^{2}$	$-4.6520\times 10^{-1}$
		$dY_{2}/dX_{1}$	$3.9785\times 10^{7}$	$3.7705\times 10^{2}$	$-2.5150\times 10^{-1}$
		$dY_{3}/dX_{1}$	$5.5873\times 10^{6}$	$1.8564\times 10^{2}$	$-1.9100\times 10^{-2}$
		$dY_{4}/dX_{1}$	$1.5940\times 10^{13}$	$5.3401\times 10^{4}$	$-4.4738\times 10^{6}$
		$dY_{5}/dX_{1}$	$2.3453\times 10^{7}$	$1.2887\times 10^{2}$	−2.5973
		$dY_{6}/dX_{1}$	$4.2323\times 10^{12}$	$1.1728\times 10^{5}$	$-1.0066\times 10^{5}$
	SR	$dY_{1}/dX_{1}$	$1.4906\times 10^{3}$	$1.9143\times 10^{1}$	$-4.5000\times 10^{-3}$
		$dY_{2}/dX_{1}$	$1.5753\times 10^{3}$	$3.9556\times 10^{1}$	$-1.4758\times 10^{2}$
		$dY_{3}/dX_{1}$	$2.7606\times 10^{3}$	$3.1546\times 10^{1}$	$-2.2000\times 10^{-3}$
		$dY_{4}/dX_{1}$	$2.8841\times 10^{5}$	$2.3984\times 10^{2}$	$-8.9000\times 10^{-3}$
		$dY_{5}/dX_{1}$	$9.4075\times 10^{4}$	$1.2370\times 10^{2}$	$-1.2100\times 10^{-2}$
		$dY_{6}/dX_{1}$	$1.2855\times 10^{4}$	$5.7653\times 10^{1}$	$-3.4000\times 10^{-2}$
	CNN	$dY_{1}/dX_{1}$	0.0000	0.0000	0.9304
		$dY_{2}/dX_{1}$	0.0000	0.0000	0.4230
		$dY_{3}/dX_{1}$	0.0000	0.0001	0.5575
		$dY_{4}/dX_{1}$	0.0000	0.0000	0.9883
		$dY_{5}/dX_{1}$	0.0000	0.0000	0.9381
		$dY_{6}/dX_{1}$	0.0000	0.0002	0.8701
	TCN Model 1 vs. Model 2	$dY_{1}/dX_{1}$	0.0000vs.0.0000	0.0000vs.0.0000	0.8171vs.0.9998
		$dY_{2}/dX_{1}$	0.0002vs.0.0000	0.0007vs.0.0000	−0.4289vs.0.9312
		$dY_{3}/dX_{1}$	0.0000vs.0.0000	0.0012vs.0.0000	0.9168vs.0.8376
		$dY_{4}/dX_{1}$	0.0000vs.0.0000	0.0001vs.0.0000	0.5592vs.0.2085
		$dY_{5}/dX_{1}$	0.0000vs.0.0000	0.0012vs.0.0000	0.7249vs.0.9980
		$dY_{6}/dX_{1}$	0.0000vs.0.0000	0.0008vs.0.0000	0.9984vs.0.9922

**Table 3:** Performance metrics for algorithms in predictions: Bold values show the best predictions for each sample size; star values denote the top Bayesian predictions.

Phase	Methods	Variable	MSE	MAE	$R^{2}$
Predictions small samples	NN 50 Epochs	$Y_{1}$	0.1653	0.3158	0.3049
		$Y_{2}$	0.0122	0.0924	0.3125
		$Y_{3}$	13.8082	3.3928	−2.7393
		$Y_{4}$	56.1014	3.7655	0.0000
		$Y_{5}$	0.1728	0.3347	0.4435
		$Y_{6}$	6.4717	1.8645	0.9132
	NN Early-Stop	$Y_{1}$	0.2739	0.3846	−0.1520
		$Y_{2}$	0.0107	0.0777	0.3965
		$Y_{3}$	3.5552	1.4563	0.0373
		$Y_{4}$	36.5446	4.7508	0.0000
		$Y_{5}$	0.0984	0.1923	0.6829
		$Y_{6}$	5.2535	1.5618	0.9296
	PINN 3 PDEs Model 1 50 Epochs	$Y_{1}$	0.4710	0.5466	−0.9808
		$Y_{2}$	0.0071	0.0552	0.5963
		$Y_{3}$	7.6201	2.1871	−1.0635
		$Y_{4}$	24.4053	4.0371	0.0000
		$Y_{5}$	0.2559	0.4373	0.1758
		$Y_{6}$	20.8505	3.7194	0.7205
	PINN 3 PDEs Model 1 Early-Stop	$Y_{1}$	0.4262	0.4646	−0.7922
		$Y_{2}$	0.0106	0.0641	0.3986
		$Y_{3}$	28.1717	4.8865	−6.6289
		$Y_{4}$	17.3826	2.9961	0.0000
		$Y_{5}$	0.1396	0.2520	0.5504
		$Y_{6}$	6.9809	1.9604	0.9064
	PINN 1 PDE Model 1 50 Epochs	$Y_{1}$	0.2242	0.3700	0.0570
		$Y_{2}$	0.0067	0.0590	0.6195
		$Y_{3}$	5.8388	1.7115	−0.5811
		$Y_{4}$	21.3012	3.1901	0.0000
		$Y_{5}$	0.0834	0.1867	0.7313
		$Y_{6}$	9.6882	2.3592	0.8701
	PINN 1 PDE Model 1 Early-Stop	$Y_{1}$	1.5982	1.0737	−5.7206
		$Y_{2}$	0.0122	0.0728	0.3071
		$Y_{3}$	18.1349	2.6480	−3.9109
		$Y_{4}$	33.7818	4.1057	0.0000
		$Y_{5}$	0.4207	0.5274	−0.3551
		$Y_{6}$	9.7196	2.2411	0.8697
Predictions small samples	PINN 1 PDE Model 2 50 Epochs	$Y_{1}$	0.2589	0.4134	−0.0888
		$Y_{2}$	0.0075	0.0560	0.5733
		$Y_{3}$	6.4928	2.0941	−0.7583
		$Y_{4}$	7.8856	2.2620	0.0000
		$Y_{5}$	0.1046	0.1953	0.6632
		$Y_{6}$	7.4993	2.2371	0.8995
	PINN 1 PDE Model 2 Early-Stop	$Y_{1}$	0.4174	0.5433	−0.7550
		$Y_{2}$	0.0065	0.0603	0.6327
		$Y_{3}$	1.5103	0.9743	0.5910
		$Y_{4}$	8.4065	2.3679	0.0000
		$Y_{5}$	0.1501	0.2841	0.5164
		$Y_{6}$	2.8607	1.0207	0.9616
	BLR 230 samples 200 tuning 1 chain	$Y_{1}$	0.3252	0.4378	−0.3674
		$Y_{2}$	0.0192	0.0846	−0.0887
		$Y_{3}$	4.4595	1.6765	−0.2076*
		$Y_{4}$	2.9796	1.4308	0.0000
		$Y_{5}$	0.3713	0.4472	−0.1958
		$Y_{6}$	53.1858	6.1492	0.2869
	PI-BLR 3 PDEs Model 1 230 samples 200 tuning 1 chain	$Y_{1}$	0.3319	0.4402	−0.3957
		$Y_{2}$	0.0195	0.0840	−0.1033
		$Y_{3}$	4.5982	1.7016	−0.2452
		$Y_{4}$	2.5773	1.3180	0.0000
		$Y_{5}$	0.3632	0.4460	−0.1698
		$Y_{6}$	48.1624	5.7915	0.3543
	BNN 10 neurons 2 hidden layers 230 samples 200 tuning 1 chain	$Y_{1}$	0.3586	0.4752	−0.5079
		$Y_{2}$	0.0183	0.0798	−0.0334
		$Y_{3}$	8.2709	2.3538	−1.2398
		$Y_{4}$	0.8176	0.8727	0.0000
		$Y_{5}$	0.3127	0.4534	−0.0072*
		$Y_{6}$	79.5339	8.1894	−0.0663
	B-PINN 3 PDEs, Model 1 10 neurons 2 hidden layers 230 samples 200 tuning, 1 chain	$Y_{1}$	0.3003	0.4290	−0.2218*
		$Y_{2}$	0.0184	0.0922	−0.0138*
		$Y_{3}$	9.9477	2.6143	−1.6404
		$Y_{4}$	1.2192	1.0707	0.0000
		$Y_{5}$	1.1319	0.9782	−2.5539
		$Y_{6}$	73.3609	8.3759	0.0012*
Predictions medium samples	NN 50 Epochs	$Y_{1}$	0.0493	0.1353	0.7925
		$Y_{2}$	0.0056	0.0463	0.6845
		$Y_{3}$	0.8598	0.7093	0.7672
		$Y_{4}$	1.3986	0.6100	0.0000
		$Y_{5}$	0.5047	0.5316	−0.6255
		$Y_{6}$	1.7182	0.6775	0.9770
	NN Early-Stop	$Y_{1}$	0.0762	0.1651	0.6798
		$Y_{2}$	0.0046	0.0372	0.7391
		$Y_{3}$	0.9166	0.7697	0.7518
		$Y_{4}$	1.1903	0.6661	0.0000
		$Y_{5}$	0.4131	0.5076	−0.3305
		$Y_{6}$	1.6525	0.6295	0.9778
Predictions medium samples	PINN 3 PDEs model 1 50 Epochs	$Y_{1}$	0.0706	0.1731	0.7031
		$Y_{2}$	0.0054	0.0462	0.6957
		$Y_{3}$	0.6627	0.6286	0.8205
		$Y_{4}$	0.6099	0.5457	0.0000
		$Y_{5}$	0.6838	0.6520	−1.2024
		$Y_{6}$	2.1924	0.7246	0.9706
	PINN 3 PDEs Model 1 Early-Stop	$Y_{1}$	0.0549	0.1535	0.7693
		$Y_{2}$	0.0041	0.0369	0.7679
		$Y_{3}$	0.5982	0.5925	0.8380
		$Y_{4}$	1.7424	0.9240	0.0000
		$Y_{5}$	1.3734	0.8931	−3.4236
		$Y_{6}$	1.8844	0.7179	0.9747
	PINN 1 PDE Model 1 50 Epochs	$Y_{1}$	0.0972	0.2356	0.5914
		$Y_{2}$	0.0050	0.0508	0.7145
		$Y_{3}$	1.0653	0.8207	0.7115
		$Y_{4}$	1.2751	0.6275	0.0000
		$Y_{5}$	0.3296	0.3793	−0.0617
		$Y_{6}$	1.9017	0.6159	0.9745
	PINN 1 PDE Model 1 Early-Stop	$Y_{1}$	0.0534	0.1545	0.7756
		$Y_{2}$	0.0038	0.0366	0.7840
		$Y_{3}$	0.5446	0.5817	0.8525
		$Y_{4}$	1.4381	0.8962	0.0000
		$Y_{5}$	0.7744	0.7441	−1.4944
		$Y_{6}$	1.4532	0.6028	0.9805
Predictions medium samples	PINN 1 PDE Model 2 50 Epochs	$Y_{1}$	0.0519	0.1485	0.7816
		$Y_{2}$	0.0041	0.0342	0.7708
		$Y_{3}$	0.5349	0.5641	0.8552
		$Y_{4}$	0.5675	0.5921	0.0000
		$Y_{5}$	0.2466	0.3829	0.2056
		$Y_{6}$	1.3562	0.4935	0.9818
	PINN 1 PDE Model 2 Early-Stop	$Y_{1}$	0.0530	0.1393	0.7770
		$Y_{2}$	0.0038	0.0354	0.7849
		$Y_{3}$	0.9487	0.7718	0.7431
		$Y_{4}$	0.7525	0.5272	0.0000
		$Y_{5}$	1.4244	1.0116	−3.5878
		$Y_{6}$	1.5919	0.5432	0.9787
	BLR 230 samples 200 tuning 1 chain	$Y_{1}$	0.3061	0.4203	−0.2872
		$Y_{2}$	0.0199	0.0857	−0.1250
		$Y_{3}$	4.5271	1.6927	−0.2259
		$Y_{4}$	3.5336	1.7652	0.0000
		$Y_{5}$	1.3282	0.9886	−3.2779
		$Y_{6}$	45.0573	5.3436	0.3959
	PI-BLR 3 PDEs Model 1 230 samples 200 tuning 1 chain	$Y_{1}$	0.3136	0.4240	−0.3186
		$Y_{2}$	0.0198	0.0863	−0.1193
		$Y_{3}$	4.4868	1.6876	−0.2150*
		$Y_{4}$	2.5198	1.4567	0.0000
		$Y_{5}$	1.3857	1.0130	−3.4632
		$Y_{6}$	44.7380	5.2786	0.4002
Predictions medium samples	BNN 10 neurons 2 hidden layers 230 samples 200 tuning 1 chain	$Y_{1}$	0.2693	0.3981	−0.1326
		$Y_{2}$	0.0177	0.0836	0.0006*
		$Y_{3}$	4.7358	1.7381	−0.2825
		$Y_{4}$	1.7934	1.3088	0.0000
		$Y_{5}$	0.8035	0.8207	−1.5880
		$Y_{6}$	75.4659	8.4365	−0.0118
	B-PINN 3 PDEs, Model 1 10 neurons 2 hidden layers 230 samples 200 tuning, 1 chain	$Y_{1}$	0.2675	0.3989	−0.1248*
		$Y_{2}$	0.0178	0.0841	−0.0043
		$Y_{3}$	4.8732	1.7617	−0.3197
		$Y_{4}$	1.7755	1.3020	0.0000
		$Y_{5}$	0.7827	0.8106	−1.5210*
		$Y_{6}$	74.7314	8.3907	−0.0019*
Predictions large samples	NN 50 Epochs	$Y_{1}$	0.0379	0.1157	0.8459
		$Y_{2}$	0.0043	0.0351	0.7655
		$Y_{3}$	0.6136	0.6095	0.8371
		$Y_{4}$	0.4036	0.6082	0.0000
		$Y_{5}$	0.6496	0.6310	−1.0394
		$Y_{6}$	1.4037	0.7219	0.9809
	NN Early-Stop	$Y_{1}$	0.0367	0.1044	0.8506
		$Y_{2}$	0.0041	0.0317	0.7749
		$Y_{3}$	0.6614	0.6423	0.8244
		$Y_{4}$	0.0658	0.1812	0.0000
		$Y_{5}$	0.2918	0.3825	0.0838
		$Y_{6}$	0.9804	0.3697	0.9867
	PINN 3 PDEs Model 1 50 Epochs	$Y_{1}$	0.0578	0.1628	0.7649
		$Y_{2}$	0.0040	0.0325	0.7784
		$Y_{3}$	0.5438	0.5614	0.8557
		$Y_{4}$	0.0778	0.1975	0.0000
		$Y_{5}$	0.2592	0.3673	0.1863
		$Y_{6}$	1.3535	0.3338	0.9816
	PINN 3 PDEs Model 1 Early-Stop	$Y_{1}$	0.0419	0.1221	0.8293
		$Y_{2}$	0.0040	0.0325	0.7795
		$Y_{3}$	0.5847	0.5921	0.8448
		$Y_{4}$	0.1018	0.2574	0.0000
		$Y_{5}$	0.2672	0.3596	0.1612
		$Y_{6}$	0.9316	0.3786	0.9873
	PINN 1 PDE Model 1 50 Epochs	$Y_{1}$	0.0457	0.1328	0.8142
		$Y_{2}$	0.0040	0.0331	0.7778
		$Y_{3}$	0.6872	0.6369	0.8176
		$Y_{4}$	0.1530	0.3383	0.0000
		$Y_{5}$	0.1562	0.2529	0.5096
		$Y_{6}$	1.3277	0.3246	0.9819
	PINN 1 PDE Model 1 Early-Stop	$Y_{1}$	0.0534	0.1547	0.7827
		$Y_{2}$	0.0039	0.0310	0.7877
		$Y_{3}$	0.6382	0.6238	0.8306
		$Y_{4}$	0.0573	0.1453	0.0000
		$Y_{5}$	0.1945	0.2983	0.3894
		$Y_{6}$	1.0142	0.2869	0.9862
Predictions large samples	PINN 1 PDE Model 2 50 Epochs	$Y_{1}$	0.0437	0.1061	0.8223
		$Y_{2}$	0.0041	0.0343	0.7759
		$Y_{3}$	0.4906	0.5483	0.8698
		$Y_{4}$	0.0524	0.1625	0.0000
		$Y_{5}$	0.3354	0.4038	−0.0531
		$Y_{6}$	0.7349	0.3176	0.9900
	PINN 1 PDE Model 2 Early-Stop	$Y_{1}$	0.0671	0.1869	0.7270
		$Y_{2}$	0.0039	0.0324	0.7842
		$Y_{3}$	0.6033	0.5940	0.8399
		$Y_{4}$	0.2176	0.2832	0.0000
		$Y_{5}$	0.6023	0.6148	−0.8910
		$Y_{6}$	1.3005	0.4777	0.9823
	BLR 230 samples 200 tuning 1 chain	$Y_{1}$	0.3052	0.4228	−0.2417
		$Y_{2}$	0.0215	0.0916	−0.1795
		$Y_{3}$	4.5046	1.6909	−0.1957
		$Y_{4}$	2.7333	1.5126	0.0000
		$Y_{5}$	1.5531	1.0503	−3.8761
		$Y_{6}$	46.8142	5.5291	0.3626
	PI-BLR 3 PDEs Model 1 230 samples 200 tuning 1 chain	$Y_{1}$	0.3074	0.4258	−0.2506
		$Y_{2}$	0.0216	0.0909	−0.1859
		$Y_{3}$	4.4982	1.6922	−0.1940
		$Y_{4}$	1.9658	1.2354	0.0000
		$Y_{5}$	1.6085	1.0786	−4.0502
		$Y_{6}$	46.7515	5.4492	0.3635
	BNN 10 neurons 2 hidden layers 230 samples 200 tuning 1 chain	$Y_{1}$	0.3045	0.4331	−0.2390
		$Y_{2}$	0.0185	0.0922	−0.0171
		$Y_{3}$	9.9584	2.6197	−1.6433
		$Y_{4}$	1.2116	1.0683	0.0000
		$Y_{5}$	1.1297	0.9758	−2.5469*
		$Y_{6}$	73.6081	8.3863	−0.0021
	B-PINN 3 PDEs, Model 1 10 neurons 2 hidden layers 230 samples 200 tuning, 1 chain	$Y_{1}$	0.3003	0.4290	−0.2218*
		$Y_{2}$	0.0184	0.0922	−0.0138*
		$Y_{3}$	9.9477	2.6143	−1.6404*
		$Y_{4}$	1.2192	1.0707	0.0000
		$Y_{5}$	1.1319	0.9782	−2.5539
		$Y_{6}$	73.3609	8.3759	0.0012*

## Data Availability

The data supporting the findings in Section 6 are openly available at https://www.kaggle.com/datasets/uciml/electric-power-consumption-data-set, accessed on 22 June 2025.

## Appendix A. SINDy Algorithm

1. Prepare the system data $X\in R^{n\times d}$ for inputs and $Y\in R^{n\times p}$ for outputs. Here, *n* represents the number of samples, *d* denotes the number of input features, and *p* indicates the number of target variables. Note that the input X contains both dynamic and state variables, while the output Y consists of the state variables of X, excluding the dynamic variables. In some applications, there might be only one dynamic variable (e.g., elapsed time *t*), which leads to the relationship d=p+1. For clarification, consider the following example:
  $$\begin{array}{cc} X & =(T\;\left(\mathrm{time}\right),X_{1}\;\left(\mathrm{temperature}\right),X_{2}\;\left(\mathrm{moisture}\right)),\\ Y & =(Y_{1}\;\left(\mathrm{temperature}\right),Y_{2}\;\left(\mathrm{moisture}\right)), \end{array}$$
  where T is the only dynamic variable.
  When dealing with MTS, it is important to account for time steps when preparing the output variables. The output variable Y should be shifted by *T* samples from the input X corresponding to the state variables, which is known as the lag operator in MTS contexts. This means that the first *T* samples of the state variables in X must be removed to construct the outputs. The larger the value of *T*, the more accurate the predictions become, but this also requires more computation. To simplify notation, assume the number of samples in both X and Y is equal to *n*.
2. Calculate the necessary derivatives $\dot{Y}\in R^{n\times (d^{\prime }\times p)}$ of the output variables with respect to the dynamic variables:
  $$\dot{Y}=\left(\frac{\partial Y}{\partial X_{1}},\ldots ,\frac{\partial Y}{\partial X_{d^{\prime }}}\right),\;\mathrm{where}\;\frac{\partial Y}{\partial X_{i}}=\left(\frac{\partial Y_{1}}{\partial X_{i}},\ldots ,\frac{\partial Y_{p}}{\partial X_{i}}\right),\;\mathrm{for}\;i=1,\ldots ,d^{\prime },$$
  where $\frac{\partial Y_{j}}{\partial X_{i}}$ for j=1,…,p is defined:
  $$\frac{\partial Y_{j}}{\partial X_{i}}=\frac{Y_{j}\left(X_{i}+\Delta X_{i}\right)-Y_{j}\left(X_{i}\right)}{\Delta X_{i}}$$
  where $d^{\prime }$ is the number of dynamic variables. In this definition, $d^{\prime }$ can be replaced by *d* to take the derivatives with respect to all inputs if desired. This may involve numerical differentiation techniques to estimate the derivatives from the collected data. In this context, $d^{\prime }\leq d$ represents the total number of dynamic variables.
3. Create a library of potential basic functions for the state variables Θ(X), including polynomial terms, interactions between variables, trigonometric functions, and other relevant functions that may describe the dynamics of the system. This step is crucial, as it lays the foundation for accurately identifying the underlying equations that govern the system’s behavior. For more complex models, one might also incorporate the derivatives of X into the potential function, resulting in Θ(X), where $X=(X,\dot{X})$.
4. Formulate the sparse regression problem $\dot{Y}=c⊙\Theta \left(X\right)$, where c is the coefficient vector to estimate, and ⊙ is the Hadamard product.
5. Regularize the coefficient vector. This step helps prevent the coefficients from becoming excessively large and promotes sparsity by shrinking some coefficients to zero. Regularization adds a penalty term to the loss function that encourages sparsity. Refine the coefficient c by minimizing
  $$\min_{c}\left(∥\dot{Y}{-c⊙\Theta \left(X\right)∥}^{2}+\lambda {∥c∥}_{1}\right),$$
  where ${∥\cdot ∥}_{1}$ and ${∥\cdot ∥}^{2}$ are the $L_{1}$ and $L_{2}$ norms, respectively.
6. Select the active terms from the regression results that have non-zero coefficients.
7. Assemble the identified terms into a mathematical model, typically in the form of a PDE.
8. Validate the constructed model against the original data and select the equations that accurately describe the data.

## Appendix B. LASSO Algorithm

1. Perform same as the SINDy Algorithm to define $\Theta \left(X\right)\in R^{n\times ((p+1)\times d)}$. In this algorithm, the derivatives are obtained not only with respect to the dynamic variables, but also for other features of the input set.
2. Formulate the LASSO regression model as
  $$\min_{c}\left(∥\dot{Y}{-c⊙\Theta \left(X\right)∥}^{2}+\lambda {∥c∥}_{1}\right).$$
3. Choose a range of λ values to test (e.g., 0.01, 0.1, 1, 10, 100).
4. Implement *k*-fold cross-validation by splitting the data into *k* subsets. Use each subset as the validation set and the remaining k−1 subsets as the training set.
5. Fit the LASSO model to the training set using the current λ.
6. Calculate the MSE or MAE on the validation set and average them across all *k* folds for each λ.
7. Using the optimal λ found from cross-validation, fit the LASSO model to the entire dataset *X* and *Y* to find c. For simplicity, one can skip Steps 3 and 4 and use a predetermined value for λ.
8. Formulate the constructed function as a PDE based on the non-zero coefficients.
9. Validate the constructed PDE against a separate dataset to ensure its predictive capability.

SYNDy and LASSO use the same criterion; the difference lies in their optimization algorithms. In SYNDy, at each iteration, only the active terms (those for which the coefficients *c* are significant, exceeding a certain threshold) are retained. In the above, the used algorithm is described.

## Appendix C. B-LASSO Algorithm

1. Follow Steps 1 to 3 in the SINDy Algorithm to prepare the data and define the design matrix.
2. Formulate the B-LASSO model as follows:
  $$Y∼N\left(c⊙\Theta \left(X\right),\sigma^{2}I_{(p+1)d}\right),$$
  $$c∼L\left(\mu_{c},\lambda \right),\;\lambda ∼G\left(\alpha_{\lambda },\beta_{\lambda }\right),\;\sigma ∼\mathrm{HC}\left(\mu_{\sigma },\sigma_{\sigma }\right),\;\sigma \geq \mu_{\sigma },$$
  where $c=(c_{1},\ldots ,c_{(p+1)d})$ represents the regression coefficients of the model, $\lambda =(\lambda_{1},\ldots ,\lambda_{(p+1)d})$ denotes the positive real regularization parameters, and σ is the model noise factor. The hyperparameters $\mu_{c}=\left(\mu_{c_{1}},\ldots ,\mu_{c_{(p+1)d}}\right)$, $\alpha_{\lambda }=\left(\alpha_{\lambda_{1}},\ldots ,\alpha_{\lambda_{(p+1)d}}\right)$, $\beta_{\lambda }=\left(\beta_{\lambda_{1}},\ldots ,\beta_{\lambda_{(p+1)d}}\right)$, $\mu_{\sigma }$, and $\sigma_{\sigma }$ can be approximated based on data. The symbols N, L, G, and HC represent the Normal, Laplace, Gamma, and Half-Cauchy distributions, respectively. For simplicity, Θ(·) can be identity function.
3. Estimate the posterior distribution using a sampling method such as MCMC. The posterior distribution of the parameters c, λ, and σ given the data Y and Θ(X) is expressed as
  P(c,λ,σ∣Y,Θ(X))∝P(Y∣Θ(X),c,σ)P(c∣λ)P(λ)P(σ).
4. Approximate the coefficients c using the posterior distribution samples:
  $$\hat{c}=E\left(c|Y,\Theta \left(X\right)\right)\approx \frac{1}{NM}\sum_{i=1}^{N}\sum_{j=1}^{M}{\hat{c}}_{ij},$$
  where ${\hat{c}}_{ij}$ represents the *i*th sample of c from the *j*th chain, *N* is the number of draws per chain, and *M* is the number of chains. In this context, the highest density interval of the coefficients can be used to measure uncertainty for a specified confidence level, such as 95%. The HDI is a Bayesian credible interval that contains the most probable values of the posterior distribution.
5. Construct PDEs based on the non-zero coefficients in the form $Y\approx \hat{c}⊙\Theta \left(X\right)$.
6. Evaluate the constructed PDEs using unseen data and select the equations that demonstrate high accuracy.

## Appendix D. SR Algorithm

1. Prepare the relevant input features $X\in R^{n\times d}$ and target features $Y\in R^{n\times p}$.
2. Compute the derivative matrix $D\in R^{n\times (d\times p)}$ of the output variables with respect to the input variables.
3. Utilize a SR package, such as gplearn (a Python library for SR based on genetic programming, for example, version 3.10.16), to approximate the relationship between the derivatives and the target variables.
4. Fit a symbolic regressor S to the training data D to approximate the relationship:
  S:D→Y.
  The symbolic regressor searches for a mathematical expression Θ that minimizes the error:
  $$\Theta^{*}=\arg\min_{\Theta }{∥Y-\Theta \left(D\right)∥}^{2}.$$
5. Construct the best performing equation $\Theta^{*}$ from the symbolic regressor S. For example, one PDE may have the form:
  $$\frac{\partial Y_{1}}{\partial X_{1}}=\Theta^{*}\left(\frac{\partial Y}{\partial X_{-1}}\right),$$
  where $Y_{1}$ is the first target feature, $X_{1}$ is the first input feature, and $X_{-1}$ represents all input features except the first one.
6. Evaluate the constructed PDEs and select those with high accuracy.

## Appendix E. CNN Algorithm

1. Provide the input features $X\in R^{n\times T\times d}$ and the target variables Y, where *T* is the number of time steps, known as lags and explained in Appendix A. For example, T=10 means that 10 previous time steps are used to predict the value at time T+1.
2. Apply one-dimensional convolutional layers with filters $W_{l}\in R^{m\times d}$ to the *l*th layer, where *m* is the kernel size. The output of the *l*th convolutional layer is
  $$H_{l}=\mathrm{ReLU}\left(W_{l}*H_{l-1}+b_{l}\right),$$
  where * denotes the convolution operation, $H_{l-1}$ is the output of the previous layer, $b_{l}$ is the bias term, and ReLU is the activation function.
3. Use max-pooling to reduce the dimensionality of the feature maps:
  $$P_{l}=\mathrm{MaxPool}\left(H_{l}\right).$$
4. Flatten the output of the final convolutional layer and pass it through a fully connected layer to produce the predicted outputs $\hat{Y}\in R^{n\times p}$:
  $$\hat{Y}=W_{L}\cdot \mathrm{Flatten}\left(P_{L}\right)+b_{L},$$
  where $W_{L}$ and $b_{L}$ are the weights and biases of the last layer and · denotes matrix multiplication.
5. Train the model using the MSE loss function. To obtain accurate derivatives, it is essential to have a well-trained network, as this ensures the reliability of the derivatives produced afterward.
6. Compute the derivatives of the predicted outputs $\hat{Y}$ with respect to the input features X using automatic differentiation:
  $$\frac{\partial \hat{Y}}{\partial X}=\nabla_{X}\hat{Y}.$$
7. Organize the derivatives into a matrix $D\in R^{n\times T\times (p\times d)}$, where each entry $D_{i,t,k}$ represents the derivative of the *j*th output variable ${\hat{Y}}_{i,j}$ with respect to the *k*th input feature $X_{i,t,k}$ at time step *t* for the *i*th sample:
  $$D_{i,t,k}=\frac{\partial {\hat{Y}}_{i,j}}{\partial X_{i,t,k}}.$$
8. Perform a correlation analysis between the derivatives and the target variables to identify the most significant terms in the PDEs. Compute the correlation matrix to quantify the relationships.
9. Select the derivatives with the highest correlation (e.g., correlation greater than 0.5) to the target variables. These derivatives are considered candidate terms for the PDEs. For example, to construct a PDE for a specific output feature with respect to a specific input feature, such as $\frac{\partial {\hat{Y}}_{1}}{\partial X_{1}}$, we select the most relevant derivatives and state variables associated with the desired derivative.
10. Approximate the relationship between the significant derivatives and the target variables using a polynomial regression model. The resulting polynomial equation represents the constructed PDE:
  $$\frac{\partial \hat{Y}}{\partial X}=\beta_{0}+\sum_{i=1}^{q}\beta_{i}\Phi_{i},$$
  where Φ represents polynomial features of degree *q* constructed from the significant derivatives and state variables.
11. Evaluate the accuracy of the constructed PDEs and select those with the highest predictive performance.

## Appendix F. TCN Algorithm

1. Follow Step 1 of the CNN Algorithm.
2. Apply a TCN to the input data. The TCN consists of multiple dilated convolutional layers with causal padding to preserve the temporal order of the data. The output of the *l*th dilated convolutional layer is given by
  $$H_{l_{d}}=\mathrm{ReLU}\left(W_{l}{*}_{r_{l}}H_{l-1}+b_{l}\right),$$
  where ${*}_{r_{l}}$ denotes the causal convolution operation with a dilation rate $r_{l}$ that increases exponentially with each layer. To ensure that the model does not utilize future information for predictions, causal padding is applied. For a kernel of size *m* and a dilation rate $r_{l}$, the input is padded with $\left(m-1\right)r_{l}$ zeros on the left side.
3. Add residual connections to improve gradient flow and stabilize training. For the *l*th layer, the residual connection is computed as
  $$h\left(H_{l-1}\right)=W_{\mathrm{res}}*H_{l-1}+b_{\mathrm{res}},$$
  where $W_{\mathrm{res}}$ and $b_{\mathrm{res}}$ are the residual weights and bias. The final output of the layer is
  $$H_{l}=H_{l_{d}}+h\left(H_{l-1}\right).$$
4. Perform Steps 4 to 11 of the CNN Algorithm.

## Appendix G. PINN Algorithm

1. Prepare a new dataset of inputs X and outputs Y, similar to Step 1 in the SINDy Algorithm.
2. Create a NN architecture aligned with the number of inputs and outputs according to the data.
3. Define the physical loss function $L_{P}$ based on the constructed PDEs. For example, if a PDE is constructed as
  $$\frac{\partial Y_{j}}{\partial X_{i}}=f\left(\frac{\partial Y}{\partial X_{-i}}\right),$$
  where f(·) is a non-linear function, $i\in \{1,\ldots ,d^{\prime }\}$, and j∈{1,…,p}, then the loss function is defined by the MSE:
  $$L_{P}=\sum_{s=1}^{n}\sum_{i=1}^{d^{\prime }}\sum_{j=1}^{p}a_{j}{∥\frac{\partial Y_{js}}{\partial X_{is}}-f\left(\frac{\partial Y_{s}}{\partial X_{-i\;s}}\right)∥}^{2},$$
  where $a=(a_{1},\ldots ,a_{p})$ is a vector of zeros and ones. The values of $a_{j}$ indicate whether the corresponding derivative equations are included in the loss function based on the PDE construction. If $a_{j}=1$, then $\frac{\partial Y_{j}}{\partial X_{i}}$ contributes to the loss function; conversely, $a_{j}=0$ indicates that no equation is defined for $\frac{\partial Y_{j}}{\partial X_{i}}$, and it is excluded from the loss.
4. Determine the data loss function $L_{D}$:
  $$L_{D}=\sum_{s=1}^{n}\sum_{j=1}^{p}{∥Y_{js}-{\hat{Y}}_{js}∥}^{2}.$$
5. Combine the physics loss and data loss into a total loss function $L_{T}$:
  (A1) $$L_{T}=L_{P}+L_{D}.$$
  Although MSE is used in this algorithm, other loss functions can also be applied.
6. Use the Adam optimizer or any other suitable optimizer to minimize $L_{T}$ with respect to the network weights and biases.
7. Train the model for a fixed number of epochs.
8. Evaluate the trained model on the validation and test sets using the total loss function. If necessary, refine or tune the hyperparameters to achieve better results.

## Appendix H. PI-BLR Algorithm

1. Prepare the input variable $X\in R^{n\times d}$ and the output target $Y\in R^{n\times p}$, similar to the SINDy Algorithm.
2. Define the priors and the Normal likelihood for the observed outputs:
  $$Y∼N\left(c⊙X,\sigma^{2}I_{p}\right),$$
  $$c=\lambda ⊙\epsilon ,\;\lambda ∼G\left(\alpha_{\lambda },\beta_{\lambda }\right),\;\epsilon ∼N\left(0,\sigma_{\epsilon }^{2}\right),\;\sigma ∼\mathrm{HC}\left(\mu_{\sigma },\sigma_{\sigma }\right),$$
  where $\sigma_{\epsilon }^{2}$, $\mu_{\sigma }$, and $\sigma_{\sigma }$ are hyperparameters. The term ϵ is included to enhance sampling efficiency, while c represents the regression coefficients with a non-centered parameterization for stable sampling. The scale parameters λ enforce sparsity through Gamma priors.
3. Specify the desired PDEs as in Step 3 of the PINN Algorithm.
4. Construct the posterior distribution of c, λ, and σ given the data X and Y:
  P(c,λ,σ∣Y,X)∝P(Y∣X,c,σ)P(c∣λ)P(λ)P(σ).
5. Set the PDEs to zero:
  $${\mathrm{PDE}}_{ji}\left(c\right)=\frac{\partial Y_{j}}{\partial X_{i}}-f\left(\frac{\partial Y}{\partial X_{-i}}\right),\;j=1,\ldots ,p,\;i=1,\ldots ,d.$$
  Note that ${\mathrm{PDE}}_{ji}$ does not need to be defined for all *j* and *i*. For undefined cases, assume it to be zero. For simplicity, assume there are $p^{\prime }$ PDEs, which can be denoted as $\mathrm{PDE}=({\mathrm{PDE}}_{1},\ldots ,{\mathrm{PDE}}_{p^{\prime }})$. The derivatives are inherently dependent on the coefficients c, although this dependence may not be explicitly clear in the preceding formula. This relationship is derived based on the model established in Step 2. The PDE residuals act as soft constraints, weighted by $\sigma_{\mathrm{PDE}}$, which can be interpreted as assuming the PDE residuals follow a zero-mean Normal distribution:
  $$\mathrm{PDE}∼N\left(0,\sigma_{\mathrm{PDE}}^{2}\right).$$
  The logarithmic term for the PDEs is given by
  $$\log P\left(\mathrm{PDE}|c\right)=-\frac{1}{2\sigma_{\mathrm{PDE}}^{2}}\sum_{i=1}^{p^{\prime }}{\mathrm{PDE}}_{i}^{2}+C.$$
  Here, logP(PDE∣c) serves as a quadratic penalty, equivalent to a Normal prior on the PDE residuals.
6. Rewrite the posterior distribution to incorporate the physics-based potential term, penalizing deviations from domain-specific PDEs:
  P(c,λ,σ∣Y,X)∝P(Y∣X,c,σ)P(c∣λ)P(λ)P(σ)P(PDE∣c).
7. Find the maximum a posteriori estimation to initialize the parameters:
  $$c_{0},\lambda_{0},\sigma_{0}=\arg\max_{c,\lambda ,\sigma }\log P\left(c,\lambda ,\sigma |Y,X\right),$$
  where $c_{0}$, $\lambda_{0}$, and $\sigma_{0}$ are the initialization parameter estimations.
8. Sample from the full posterior using the No-U-Turn Sampler (NUTS) and specify appropriate values for the number of chains, tuning steps, number of returned samples, target acceptance rate, and maximum tree depth. It is worth mentioning that tuning steps, also known as burn-in, refer to the number of initial samples to discard before collecting the desired number of samples from each chain.
9. Evaluate the model on an unseen dataset and adjust the hyperparameters as necessary.

## Appendix I. B-PINN Algorithm

1. Start by following Step 1 of the SINDy Algorithm to load the input and output datasets.
2. Define the initial layer and subsequent layers using a non-centered parameterization of weights:
  $$\begin{array}{cc} H_{1} & =\tanh(X\left(cW_{1}\right)+b_{1}),\\ H_{l} & =\tanh(H_{l-1}\left(cW_{l}\right)+b_{l}),\;l=2,\ldots ,L-1,\\ \hat{Y} & =H_{L-1}\left(cW_{L}\right)+b_{L}, \end{array}$$
  where *c* is a small scalar, such as 0.01, to ensure the weights are non-centered, and *L* represents the total number of layers. All weights $W=(W_{1},\ldots ,W_{L})$ and biases $b=(b_{1},\ldots ,b_{L})$ are assumed to follow distributions $N(0,\sigma_{W}^{2})$ and $N(0,\sigma_{b}^{2})$, respectively, with typical values being $\sigma_{W}^{2}=1$ and $\sigma_{b}^{2}=0.1$. The hyperbolic tangent function is omitted in the last layer for regression tasks.
3. Assign a Half-Normal prior to the observational noise, represented as $\sigma ∼\mathrm{HN}(\sigma_{\sigma })$, where HN denotes the Half-Normal distribution. This prior, being lighter-tailed compared to Half-Cauchy, enhances identifiability in high-dimensional B-PINNs by penalizing large noise values.
4. Construct the likelihood function based on the prediction $\hat{Y}\in R^{n\times p}$:
  $$Y∼N(\hat{Y},\sigma^{2}I_{p}).$$
5. Formulate the PDEs as outlined in Steps 3 and 5 of the PI-BLR Algorithm.
6. Define the hierarchical Bayesian inference using either the PDEs or by omitting their current term to formulate a BNN:
  $$\begin{array}{cc} P(W,b|Y,X)\propto & \underset{\mathrm{Likelihood}}{\underset{︸}{P(Y,X|W,b)}}\underset{\mathrm{Prior}}{\underset{︸}{P(W)P(b)}}\underset{\mathrm{Physics}}{\underset{︸}{P(PDE|W,b)}} \end{array}$$
7. Use Automatic Differentiation Variational Inference (ADVI) to approximate initial points for the parameters. ADVI is a type of variational inference aimed at approximating the true posterior distribution by a simpler, parameterized distribution, achieved by minimizing the Kullback–Leibler divergence between the two distributions.
8. Sample from the approximated posterior distribution using NUTS with the hyperparameters defined in Step 8 of the PI-BLR Algorithm.
9. Evaluate the trained model to determine its reliability.
